# Energy Transfer in Aqueous Light Harvesting Antennae Based on Brush-like Inter-Conjugated Polyelectrolyte Complexes

**DOI:** 10.1021/acs.macromol.2c01291

**Published:** 2022-11-29

**Authors:** Gregory M. Pitch, Levi N. Matsushima, Yannick Kraemer, Eric A. Dailing, Alexander L. Ayzner

**Affiliations:** † Department of Chemistry and Biochemistry, University of California Santa Cruz, Santa Cruz, California95064, United States; ‡ The Molecular Foundry, Lawrence Berkeley National Laboratory, Berkeley, California94720, United States

## Abstract

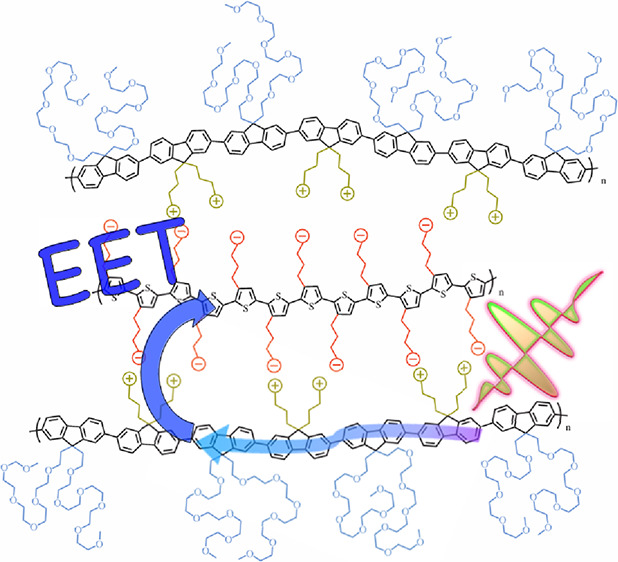

Conjugated polyelectrolytes (CPEs) have the potential to serve as building blocks of artificial light-harvesting systems. This is primarily due to their delocalized electronic states and potential for hierarchical self-assembly. We showed previously that inter-CPE complexes composed of oppositely charged exciton–donor and exciton–acceptor CPEs displayed efficient electronic energy transfer. However, near ionic charge equivalence, complexed CPE chains become net-neutral and thus experience a precipitous drop in aqueous solubility. To increase the stability and to rationally manipulate the phase behavior of inter-CPE complexes, we synthesized a series of highly water-soluble exciton–donor CPEs composed of alternating ionic and polar nonionic fluorene monomers. The nonionic monomer contained oligo(ethyleneglycol) sidechains of variable length. We then formed exciton donor–acceptor complexes and investigated their relative energy transfer efficiencies in the presence of a fixed exciton–acceptor CPE. We find that, even when the polar nonionic sidechains become quite long (nine ethyleneglycol units), the energy transfer efficiency is hardly affected so long as the inter-CPE network retains a net polyelectrolyte charge. However, near the onset of spontaneous phase separation, we observe a clear influence of the length of the oligo(ethyleneglycol) sidechains on the photophysics of the complex. Our results have implications for the use of polyelectrolyte phase separation to produce aqueous light-harvesting soft materials.

## 1 Introduction

Constructing complex artificial light-harvesting systems that can mimic the core aspects of natural light-harvesting organelles is a chemical grand challenge.^1^ At its foundational level, such a system should exhibit panchromatic light absorption and the ability to efficiently move electronic excited states (excitons) through space.^2^ Exciton migration increases the probability that photon energy will eventually be converted to chemical potential energy of spatially separated electron–hole pairs.^3−5^

It is desirable to be able to construct such an artificial light-harvesting system in water—the most environmentally benign medium. This imposes significant constraints. One constraint is the need to impart aqueous solubility to molecular semiconductors that possess the propensity for hydrophobic and π-stacking interactions^6−12^ as well as the associated need to ensure that their hierarchical assemblies are stable. Among examples that satisfy these requirements, one elegant solution is an aqueous light-harvesting antenna developed by the Tovar group. Therein, small-molecule organic semiconductors decorated with peptidic sidechains were used to build in specific aggregation motifs while retaining sufficient aqueous solubility. π-Stacking interactions between adjacent organic semiconductors within the assembly gave rise to an electronic coupling and resultant exciton motion down the stacking axis.^13,14^

Our group has shown that electrostatic assemblies of conjugated polyelectrolytes (CPEs), water-soluble semiconducting polymers,^15−20^ hold substantial promise as primary building blocks of complex aqueous light-harvesting systems. First, CPEs support highly delocalized electronic states, which leads to rapid intrachain exciton motion over distances that are large compared to the monomer length.^21^ Second, oppositely charged CPEs that function as an exciton donor–acceptor pair may be readily electrostatically assembled to form artificial light-harvesting antennae that exhibit ultrafast (sub-250 fs) exciton transfer.^22^ Finally, and crucially, we argue that the associative phase separation of aqueous polyelectrolyte assemblies provides a tractable and relatively simple pathway to complex, multi-component, and multi-functional light-harvesting systems. We recently showed that associative phase separation of one conjugated and one non-conjugated polyelectrolyte allowed us to form colloidal gels with tunable photophysical properties that were sensitive to specific cation-π interactions.^23^ We also showed that phase separation of exciton donor–acceptor CPEs leads to the formation of complex fluids with highly efficient exciton transfer and mechanical properties that can be tuned using the ionic atmosphere.^24^

Of particular interest is the formation of a liquid coacervate phase, which is a dense, highly polyelectrolyte-enriched liquid.^25−29^ Forming such a coacervate via phase separation from oppositely charged CPEs that function as an exciton donor–acceptor pair would constitute a major step toward light-harvesting soft-matter complexity. Such coacervates could function as photophysically active compartments in a larger overarching system or as fluid exciton-transferring scaffolds for an artificial electron transport chain and molecular catalysts.

To the best of our knowledge, formation of such a truly liquid CPE-based *complex* coacervate has not yet been observed. The hypothesized viscoelastic, CPE-based complex coacervate is to be distinguished from complex fluids with a substantial solid fraction or colloidal gel states. Unlike solid-like states, the CPE coacervate is expected to exhibit temporal fluctuations in local electronic properties associated with liquid-state dynamics. We hypothesize that the difficulty in realizing a CPE coacervate is because associative phase separation is most often induced at ionic stoichiometry of oppositely charged polyelectrolytes. With CPEs, this leads to a net-neutral complex with many hydrophobic and potentially π-stacking interactions. Often the result is a solid- or gel-like assembly. Our central hypothesis is that fluidity and stability must come from polar-but-nonionic sidechains that do not directly participate in ionic inter-CPE complexation. To increase the aqueous solubility of a net-neutral inter-CPE complex, we synthesized a chemical series of polyfluorene-based CPEs with one ionic monomer and one co-monomer bearing oligo(ethyleneglycol) (oEG) sidechains with three, six, and nine units. We verified that these brush-like CPEs were highly water-soluble, with the nine-oEG derivative having a solubility in excess of 100 mg/mL. We interrogated the structure in isolated solution using a combination of light and X-ray scattering methods. We then formed oppositely charged artificial light-harvesting antennae using the synthesized exciton–donor CPE series and a polythiophene-based exciton acceptor CPE that was common to all the exciton donors. We find that the efficiency of electronic energy transfer from the donor to the acceptor CPE effectively does not decrease across the oEG length series at compositions far from nominal polyelectrolyte neutrality. This illustrates that the CPE microstructure in the complex is such that the long brush-like sidechains do not present any appreciable steric hindrance to alignment of CPE transition dipole moments. However, we show that near the onset of associative phase separation the energy transfer efficiency does in fact depend on oEG length. Our observations have implications for the construction of light-harvesting complex fluids based on CPEs.

## 2 Experimental Methods

### 2.1 Polymer Synthesis

**Figure 1:**
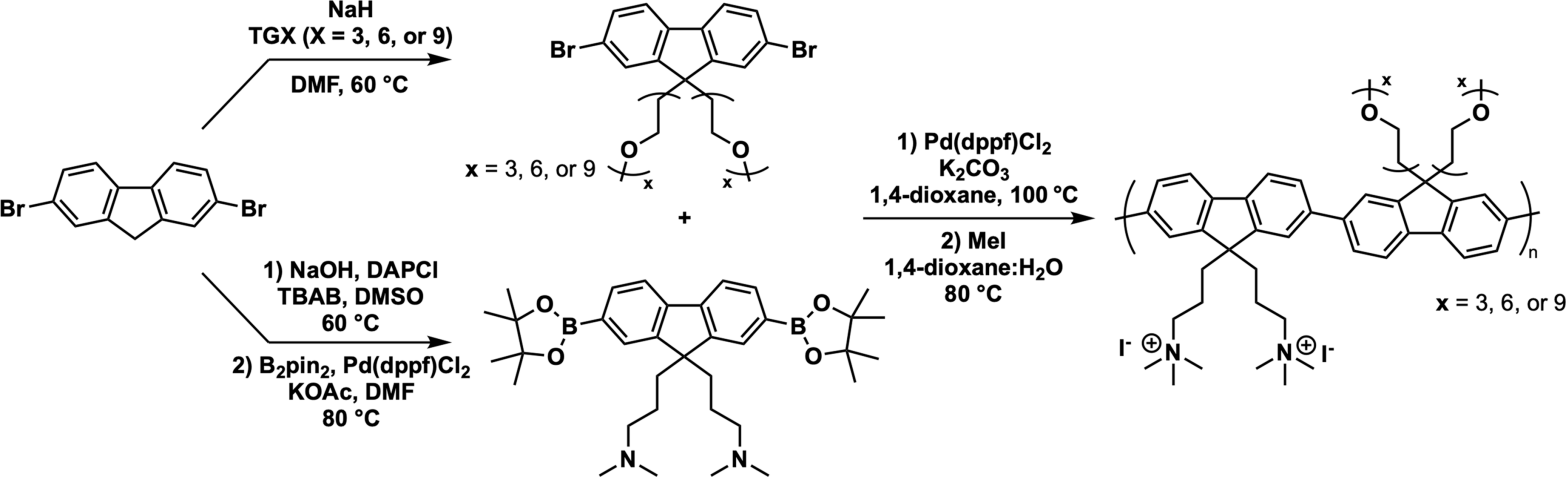
Synthetic scheme for the PFNG*X* chemical series. The dibromofluorene monomer F is converted to either its oEG derivative FG*X* or the diamine-containing monomer FN. Upon borylation of FN to form FNB, the neutral precursor polymer series nPFNG*X* is made using Suzuki coupling. The final CPE series is obtained by reacting nPFNG*X* with methyl iodide.

The full synthetic details and characterization are provided in the Supporting Information. Briefly, a polyfluorene-based exciton–donor CPE series varying in the length of oEG side chains (PFNG*X*, *X* = 3, 6, or 9) was synthesized, as shown in Figure 1. PFNG*X* was formed via Suzuki polycondensation reactions between FG*X* (*X* = 3, 6, or 9) and FNB followed by quaternization of pendant alkyl amines on the FN monomer and dialysis using a membrane with a 10,000 MW cutoff. Purity of synthesized products was evaluated with proton (^1^H) and carbon (^13^C) nuclear magnetic resonance.

### 2.2 Sample Preparation

The exciton acceptor polymer, regioregular poly[3-(potassium-2-ethanoate)thiophene-2,5-diyl] (PTAK; *M*_w_ = 16 kDa, PD = 2.2), was purchased from Rieke Metals and used as received. Stock solutions PFNG*X* were prepared at 3 mg/mL in HPLC-grade water (Sigma-Aldrich) and degassed with Ar(g). Stock solutions of PTAK were prepared at 1 mg/mL in the same manner as the PFNG*X* solutions. Solutions were wrapped in Al foil, stirred at 650 rpm, and heated at 70 °C for 6 h. Upon cooling to room temperature, stock solutions were filtered through 450 nm PVDF filters. Dilute solutions of exciton–donor CPE controls and donor–acceptor PFNG*X*:PTAK complexes were prepared in 1.5 mL natural microcentrifuge tubes (Seal Rite), wrapped in Al foil, stirred at 650 rpm, and heated at 70 °C in a sand bath for 6 h. Inter-CPE complexes were prepared in plastic centrifuge tubes as opposed to glass vials to minimize errors in the concentration in dilute spectroscopy measurements due to the ready adhesion of PFNG*X* to vial walls. The monomer concentration of PFNG*X* solutions was held constant at 1.33 × 10^–5^ M. In PFNG*X*:PTAK complex solutions, we used the following number densities of anionic PTAK sidechains relative to cationic PFNG*X* sidechains: 25, 50, and 75%. Thus, in the 75% complex solution, 75% of the total cationic PFNG*X* charge is compensated by the total anionic PTAK charge. These complex solutions corresponded to PTAK monomer concentrations of 6.65 × 10^–6^, 1.33 × 10^–5^, and 2.00 × 10^–5^ M, respectively.

### 2.3 Steady-State Spectroscopy Measurements

Optical density measurements were collected from the above solutions using a UV-2700 Shimadzu spectrometer. Spectra were collected over the 300–800 nm wavelength range in 1.0 nm increments. All spectra were measured with a 1 mm path length quartz cuvette. Photoluminscence (PL) and PL excitation (PLE) spectra were collected using a Horiba Fluoromax-4 spectrometer in a right-angle geometry. In PL measurements, the excitation wavelength was set to 375 nm, and the PL intensity was collected in the 350–800 nm range in 1 nm increments, with excitation and emission slit widths set to 1 nm bandpass. For PLE measurements, the fixed emission wavelength was set to 680 nm, and the excitation wavelength was scanned from 300 to 800 nm in 1 nm increments, with excitation and emission slit widths set to 2 nm bandpass. For quantitative quenching measurements, samples were excited with a 375 nm pulsed picosecond diode laser (BDS-SM series, Becker & Hickl, GmbH), and PL spectra were collected using a Pixis 100 CCD (Princeton Instruments) mounted on a monochromator (Acton Spectra Pro 2300, Princeton Instruments).

### 2.4 Time-Resolved Photoluminescence Spectroscopy

Time-resolved PL measurements were carried out using the time-correlated single-photon counting on a home-built setup, which has been previously described.^22^ Briefly, samples were excited at 375 nm by a pulsed picosecond diode laser (BDS-SM Series, Becker and Hickl, GmbH), and emission was measured on a hybrid photomultiplier tube (Becker and Hickl, GmbH). The signals were then sent to a Simple Tau SPC-130 (Becker and Hickl, GmbH) for initial data visualization and analysis. A high-pass filter was used on the detection arm with a 400 nm onset, while the monochromator was set to collect emission intensity at 475 nm. The samples were loaded into a front-face cuvette, while the excitation and detection polarizers were offset from each other by 54.7° to minimize polarization effects. FluoroTools DecayFit software, written by Dr. Soren Preus (Fluorescence Decay Analysis Software 1.3, FluorTools, www.fluorotools.com), was used to conduct forward convolution with a measured instrument response function to determine excited-state lifetimes. Data was fit using a non-linear least squares minimization, and final goodness of fit was evaluated via *X*^2^ values.

### 2.5 Small-Angle X-ray Scattering

Solution small-angle X-ray scattering (SAXS) was conducted at the Stanford Synchrotron Radiation Laboratory (SSRL) beamline 4-2. Samples were transferred via an autosampler from a 96-well plate into a thin-walled quartz capillary tube where they were exposed to the X-ray beam. Samples were irradiated at a photon energy of 11 keV, and scattering was collected with a Dectris PILATUS3 X 1M at a sample-detector distance of 1.7 m. Each sample was irradiated for 16 × 1 s exposures. The intensities of these images were azimuthally averaged and plotted against scattering vector length *q* after being background-subtracted via SAXSPipe software using a measured background solvent solution between each sample series. Fitting of SAXS curves followed a previously described method.^24^ Briefly, reduced SAXS curves were smoothed in the high-*q* region to reduce the influence of artifacts in the pair-distance distribution function (PDDF). To calculate the PDDF, the PCG software suite was used.

### 2.6 Dynamic Light Scattering

Dynamic light scattering (DLS) was used to measure diffusion coefficients of the charged polymer series in salt-free solutions using an adjustable angle single-detector light scattering system (BI-200SM, Brookhaven Instruments Corporation). DLS was carried out using a 637 nm diode laser (CW Mini L-30, Brookhaven Instrument Corporation), and the scattered intensity was collected at scattering angles of 45, 67, 90, 112, and 135° on an avalanche photodiode (BI-APDX, Brookhaven Instrument Corporation) on an adjustable goniometer (BI-200SM, Brookhaven Instrument Corporation). Samples were filtered through a 0.45 μm nylon fiber filter and then loaded into 12 mm borosilicate glass tubes, which were submerged in a vat of decahydronaphthalene to reduce stray scattered light by matching the refractive index of the glass sample tubes. Signals collected on the avalanche photodiode were sent first to a correlator (TurboCorr, Brookhaven Instrument Corporation), and the correlated intensities were then used to calculate the autocorrelation function. CONTIN analysis was carried out in Matlab, where the regularization parameter (α) was set to 0.1. It was found that different values for point density and α returned similar relaxation times; thus, α was kept low to not overly smooth the relaxation time distribution.

### 2.7 Size Exclusion Chromatography

Absolute molecular weight calculations were performed prior to quaternization of the neutral polymer via triple-detection size exclusion chromatography (SEC) using a Malvern OmniSEC equipped with refractive index, light scattering, and intrinsic viscosity detectors calibrated with a single polystyrene standard. Analysis was performed in tetrahydrofuran (THF) running at 1 mL min^–1^ and 35 °C.

Using the weight-average molecular weights in Table 1 and a fluorene monomer length of 6.85 Å gives the following degree of polymerization (DP, in total monomer units) and contour length *l*_c_ estimates for PFNG6 (DP ≈ 26, *l*_c_ ≈ 18 nm) and PFNG9 (DP ≈ 28, *l*_c_ ≈ 20).

**Table 1:** Polymer Molecular Weights

sample	*M*_n_(g/mol)	*M*_w_(g/mol)	*M*_w_/*M*_n_
nPFNG6	12,700	16,550	1.30
nPFNG9	16,030	23,880	1.49

The neutral precursor polymer nPFNG3 was found to be insoluble in THF or 1,2,4-trichlorobenzene at 135 °C, the latter precluding analysis via high-temperature SEC. We then attempted to measure the molecular weight by measuring angle- and concentration-dependent static light scattering to construct a Zimm plot. We found that some of the Zimm plots were (reproducibly) highly nonclassical compared to common polymers such as polystyrene. Additionally, the extrapolation to zero scattering angle and concentration led to an unphysically large *M*_w_. Since the average molecular weights and polydispersities of nPFNG6 and nPFNG9 are similar, and since all polymerization and quaternization conditions were identical, we believe that it is reasonable to assume that nPFNG3 also has a similar average molecular weight and polydispersity.

## 3 Results

Figure 1 shows a summary of our synthetic scheme and the chemical structures of our CPE chemical series. Detailed synthesis and characterization are described in the Supporting Information. The CPEs are highly water-soluble; the solubility for PFNG9 > 100 mg/mL. For reference, a related common CPE composed of a fluorene monomer with identical charged sidechains alternating with an unfunctionalized phenyl monomer forms a hydrogel at 10 mg/mL.^24^ Below, we refer to the polymers via the abbreviation PFNG*X*, where *X* corresponds to the number of ethylene glycol units on the nonionic monomer sidechain. We first discuss absorption and photoluminescence (PL) spectroscopy measurements. We then go on to interrogate the solution structure using a combination of small-angle X-ray scattering (SAXS) and angle-dependent dynamic light scattering (DLS) experiments. Finally, we interrogate electronic energy transfer (EET) between exciton–donor PFN*X* polymers and an oppositely charged polythiophene-based CPE, which serves as the common exciton acceptor.

### 3.1 Photophysics of Aqueous CPE Solutions

**Figure 2:**
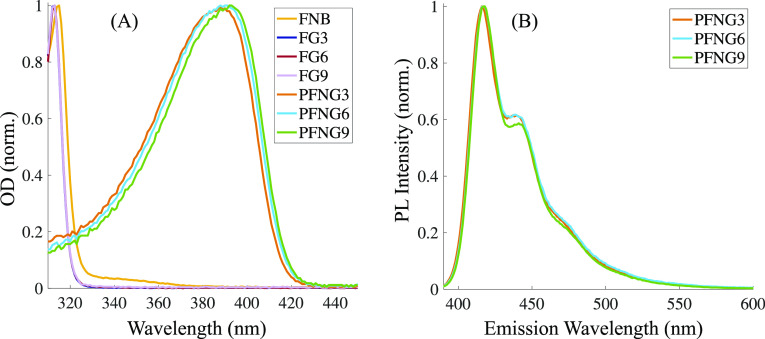
(A) Normalized OD of monomers required for the synthesis of PFNG*X* CPEs as well as normalized OD of PFNG*X* CPEs. (B) Normalized PL intensities for PFNG*X* CPEs. The excitation wavelength corresponds to the peak of the OD.

Figure 2A shows the normalized optical density (OD) of each monomer and its corresponding CPE in aqueous solution. FG*X* labels oEG-containing dibromofluorene monomers, while FNB represents the pinacol boryl ester of the dimethylaminopropyl fluorene monomer. All monomers absorb in the UV and have very similar absorption spectra. As expected, PFNG3, PFNG6, and PFNG9 display strongly redshifted spectra compared to the monomers, with λ_max_ values of 388, 391, and 393 nm, respectively. There is a subtle but monotonic spectral redshift as a function of oEG length, which suggests that the mean chromophore length is slightly longer for each successive polymer. Figure 2B shows the peak-normalized PL spectra for the three polymers following excitation at 375 nm. The position of the emission maximum is unchanged across the CPE series. The ratio of the 0–0 and 0–1 vibronic peaks, *I*_00_/*I*_01_, for PFNG9 is ∼5% larger than the other two CPEs.

**Figure 3:**
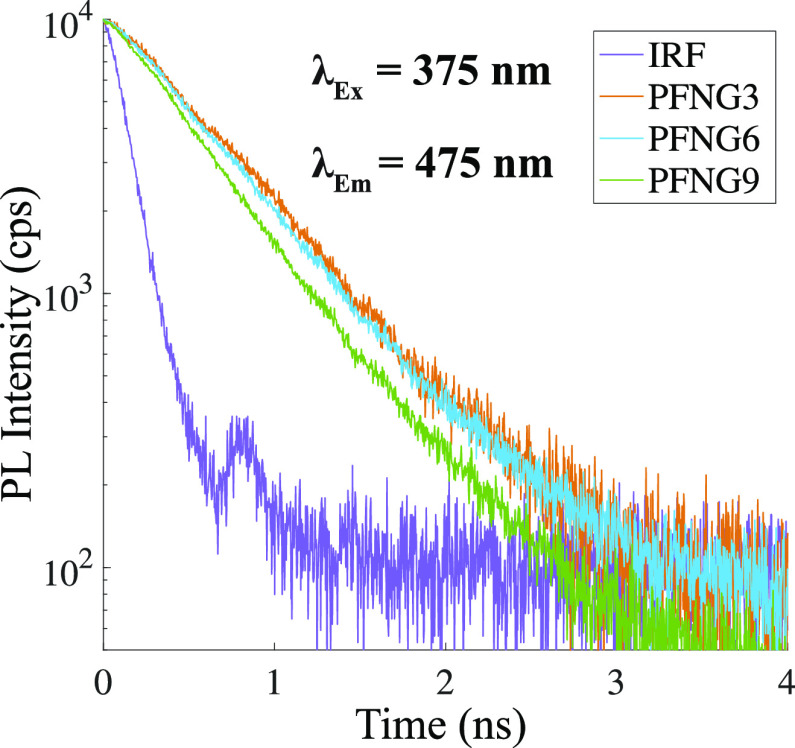
Time-resolved PL decays for PFNG*X* series. The total monomer concentration was 1.33 × 10^–5^ M. The excitation wavelength was 375 nm, and the emission wavelength was 475 nm.

Figure 3 shows time-resolved PL decays for the polymer series following excitation near the peak of the OD spectrum, along with the instrument response function (IRF). The deconvolved decays are biexponential with one dominant PL lifetime component. To compare the overall PL decays, we calculated intensity-weighted average PL lifetimes ⟨τ⟩ according to
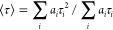
1where *a_i_* is the amplitude of component *i*, and τ_*i*_ is its decay time. We find that ⟨τ⟩ = 1.12, 0.73, and 0.63 ns for PFNG3, PFNG6, and PFNG9, respectively. The decrease in ⟨τ⟩ with oEG length is likely due to changes either in (i) the average conformation of a single chain from more extended to more coiled or (ii) the formation of higher-order structures like π-stacked assemblies.

### 3.2 CPE Microstructure in Salt-Free Aqueous Solutions

**Figure 4:**
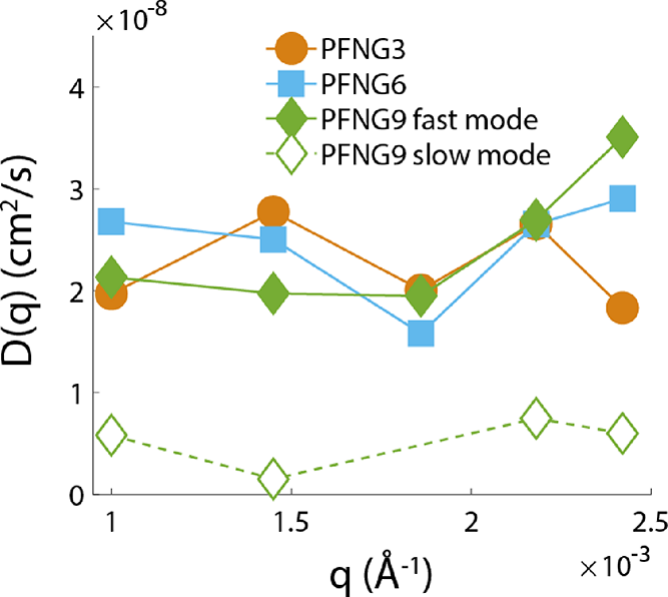
Diffusion coefficients of salt-free CPE solutions extracted from DLS autocorrelation functions using CONTIN analysis as a function of scattering vector length. PFNG9 solutions displayed both a fast (solid diamonds) and a slow (open diamonds) diffusive mode.

Having characterized the photophysical properties of CPE solutions, we proceeded to interrogate the structural characteristics of the polyelectrolyte chains in salt-free aqueous solutions. Given the smallness of the solution OD, we believe that in control donor or acceptor solutions, CPE chains are well separated from each other, corresponding to the dilute regime. We began by measuring diffusion dynamics using angle-dependent dynamic light scattering (DLS). Measurements as a function of scattering angle allow us to interrogate the dependence of the effective diffusion coefficient *D*_eff_ on the scattering vector length *q* at small wave numbers of order 10^–3^ Å^–1^. It has been pointed out that the effective diffusion coefficient for polyelectrolyte solutions may be written as
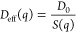
2where *D*_0_ is the so-called free-particle diffusion coefficient and *S*(*q*) is the static structure factor of the polyelectrolyte solution.^30,31^Figure 4 shows that for PFNG3 and PFNG6, *D*_eff_(*q*)∼*D*_0_, which lies between 2 × 10^–8^ and 3 × 10^–8^ cm^2^/s. PFNG9 also displays a similar diffusive mode, but we have found evidence for the presence of an additional slower mode with *D*_0_ ∼5 × 10^–9^ cm^2^/s for this CPE. A representative example of a scattering intensity autocorrelation function for PFNG9 displaying two distinct decay modes is shown in Figure S29 of the Supporting Information. Thus, it appears that the PFNG9 solution likely contains a coexistence of single CPE chains (dressed by their counterion clouds) as well as larger particles.

**Figure 5:**
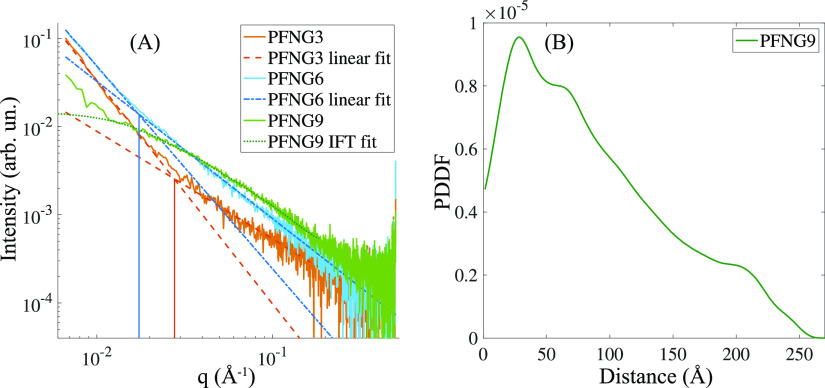
Small-angle X-ray scattering (SAXS) of CPE solutions. (A) Reduced SAXS intensities as a function of scattering vector length *q*. Sloped lines indicate fits to the linear regions, and vertical lines correspond to the intersection point of low-*q* and high-*q* linear fits. The green dotted line shows the fit obtained using the indirect Fourier transform (IFT). (B) Pair-distance distribution function (PDDF) obtained in a model-independent manner using the IFT.

To probe the solution structure further, we performed small-angle X-ray scattering measurements on the salt-free CPE solutions, the results of which are shown in Figure 5. Figure 5A shows the reduced scattering intensities as a function of *q* on a double-logarithmic scale for aqueous solutions of the PFNG*X* polymers at the same molar monomer concentration. PFNG3 and PFNG6 solutions have qualitatively similar curves. The monotonically decreasing intensity shows two linear regions, each with a different slope corresponding to a different power law exponent. For PFNG3, the low-*q* and high-*q* regions have log–log slopes of −2.53 and −1.22, respectively, with the intersection point q* = 0.0275 Å^–1^. For PFNG6, the low-*q* and high-*q* regions have slopes of −2.30 and −1.55, respectively, with the intersection point q* = 0.0171 Å^–1^. A high-*q* slope of approximately −1 is indicative of rod-like scaling.^32^ The q* that corresponds to a transition from approximately −2 to approximately −1 scaling can be used to crudely estimate the polymer persistence length *l*_p_ via q*l_p_∼ 3.5.^33^

This estimate gives *l*_p_ for PFNG3 and PFNG6 of ∼13 and ∼21 nm, respectively. Although these crude values are likely overestimates, the relative change is likely meaningful. Previous estimates of neutral alkyl-substituted polyfluorenes gave *l*_p_ ∼10 nm.^34,35^ The potential increase in *l*_p_ for our alternating polyfluorene CPEs could be justified based on sidechain repulsion arguments in the absence of excess salt. At the same time, the backbone of the conjugated polymer is largely hydrophobic, and in the absence of ionic sidechains, the solubility of the polymer would be extremely low. Addition of highly solubilizing oEG sidechains is expected to lead to increased solubility. We expect that the quality of the solvent for the polymer should be a monotonically increasing function of oEG sidechain length, at least for sidechain lengths used in this investigation. This expectation is also consistent with the apparent increase in the persistence length with increasing oEG length that we observe.

The SAXS curve for PFNG9 at low *q* does not qualitatively resemble those of PFNG3 and PFNG6. At *q* ∼0.01 Å^–1^, the scattering intensity displays a Guinier-like plateau before increasing further at lower *q*. Thus, although we performed linear fits to both the low-*q* and high-*q* regions (slopes of −1.95 and −1.37, respectively), we did not attempt to extract an *l*_p_ estimate. Instead, the appearance of a Guinier-like plateau led us to use the indirect Fourier transform (IFT) to fit the scattering curve over the *q* range above the low-*q* linear region.^36,37^ There is an underlying assumption that the scattering intensity around the Guinier plateau is due to scattering from isolated particles and not a structured assembly of particles, which is supported by the observation that *D*_eff_(*q*)∼*D*_0_. Under this assumption, we can extract the pair-distance distribution function (PDDF) for PFNG9 in a model-independent manner, which is shown in Figure 5B. We do not attempt to overinterpret the fine structure of the PDDF. Instead, we note that the general shape is consistent with a quasi-cylindrical particle,^38^ and the distance at which the PDDF goes to zero can be approximately associated with the size of the scattering inhomogeneity. This gives a value of ∼25 nm. The fact that PFNG9 seems to preferrentially form aggregated states is somewhat counterintuitive. We speculate that this arises from micellization of the PFNG9 polymer, suggesting that there may be a critical length of the oEG sidechain beyond which the micelle state is stabilized.

### 3.3 Energy Transfer in PFNG*X* Inter-CPE Complexes

With structural and photophysical characterization of isolated PFNG*X* solutions in hand, we went on to elucidate the influence of the increasing oEG sidechain length on EET in inter-CPE complexes (CPECs). To do so, we formed CPECs composed of the cationic PFNG*X* and an anionic poly(butylcarboxythiophene) CPE (PTAK).^21^ Given the spectral overlap between the OD spectrum of PTAK and the PL spectrum of PFNG*X*, in the CPEC, the PFNG*X*s serve as exciton donors while PTAK serves as the exciton acceptor. To evaluate whether PFNG*X* excitons were undergoing EET to PTAK, we fixed the donor polymer concentration at 1.33 × 10^–5^ M and varied the PFNG*X*:PTAK ionic charge ratio from 1:0.00 (donor control) to 1:0.75. We have found the phase separation point to lie in proximity of the 1:1 net polycation/polyanion charge ratio for all CPEs. In this investigation our primary goal was to study the behavior of soluble complexes that could be accurately interrogated using solution spectroscopy. Since in solutions without excess salt solid precipitates form at the 1:1 donor–acceptor CPE ratio, we have specifically limited the CPE charge ratio to fall below this value.

**Figure 6:**
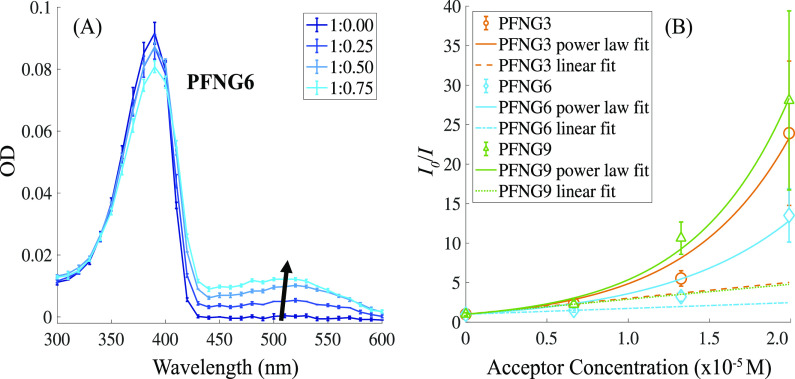
Steady-state spectroscopy of PFNG*X*:PTAK complex solutions. (A) Optical densities of isolated PFNG6 (1:0.00) and PFNG6 CPECs formed with PTAK at molar charge percentages of 25, 50, and 75%. (B) Stern–Volmer analysis for PFNG*X* CPEs: PFNG3 (orange circles), PFNG3 power law fit (orange-solid line), PFNG3 linear fit (orange-dashed line), PFNG6 (blue diamonds), PFNG6 power law fit (blue-solid line), PFNG6 linear fit (blue-dotted dashed line), PFNG9 (green triangles), PFNG9 power law fit (green-solid line), and PFNG9 linear fit (green-dotted line). Error bars correspond to one standard deviation.

Figure 6A shows the OD spectra of PFNG6 in isolation as well as with increasing PTAK concentration. CPEC absorption spectra show a characteristic peak for each component of the complex. The relatively narrow band near 390 nm corresponds to PFNG6, while the broader peak centered about 520 nm corresponds to PTAK. As expected, the PTAK OD increased monotonically with increasing PTAK concentration, which is indicated with the upward-facing arrow. The fact that the PFNG6 OD undergoes a mild decrease despite its concentration being fixed is most likely due to a small decrease in the PFNG6 extinction coefficient. This is not entirely surprising as complexation to PTAK may cause a small structural deformation of the PFNG*X* chain, leading to a decrease in the mean conjugation length. There may well be a correspondingly mild spectral shift that accompanies the change in the magnitude of the PFNG6 peak OD. However, we believe that, when added on top of the PTAK absorption band, such a shift would be challenging to discern. The corresponding OD spectra for PFNG3 and PFNG9 CPECs are shown in the Supporting Information in Figure S30. The molar concentration of each donor polymer was dilute enough such that its OD was kept below 0.1 to ensure reliability of PL quenching measurements.

We find that the PL intensity of donor CPEs decreases monotonically as the acceptor CPE concentration increases. This observation is consistent with EET from PFNG*X* to PTAK. To quantify the extent of donor PL quenching, we formed Stern–Volmer plots by plotting the ratio of the PL intensity of the unquenched donor CPE control, *I*_0_, to the intensity of the donor in the presence of the quencher, *I*, as a function of acceptor concentration.^39−42^ When the decrease in PL intensity is largely due to the formation of a single static complex, *I*_0_/*I* is often linear in the quencher concentration. The Stern–Volmer constant obtained from the slope is then a quantitative measure of quenching efficiency. Figure 6B shows Stern–Volmer plots for the three exciton–donor CPEs as a function of molar PTAK concentration. The plotted donor PL intensity values were taken at 419 nm, which corresponds to the maximum of the PFNG*X* PL spectrum. It is clear that *I*_0_/*I* departs from linearity for all three PFNG*X* derivatives over this range of donor–acceptor molar charge ratios. To quantify this behavior, we were able to approximately capture the functional form of the intensity ratio over this acceptor concentration range using a simple modified power law according to
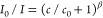
3where *c* is the acceptor concentration, *c*_0_ is the unit concentration, and β is the sole fitting parameter. We find that β are 1.58 × 10^5^, 1.28 × 10^5^, and 1.68 × 10^5^ for PFNG3, PFNG6, and PFNG9, respectively.

Nevertheless, at low acceptor concentrations *I*_0_/*I* is locally linear. To get a crude estimate of the quenching efficiency in the limit of low molar charge ratios, we also fit the low-*c* region to a linear form to extract the Stern–Volmer constant, *K*_SV_, in the limit of vanishing acceptor concentration. Estimates for  for PFNG3, PFNG6, and PFNG9 are 2.03 × 10^5^, 7.34 × 10^4^, and 1.90 × 10^5^ M^–1^, respectively. The corresponding donor PL quenching spectra for PFNG3, PFNG6, and PFNG9 CPECs are shown in the Supporting Information in Figure S31.

Although the quenching of donor PL by the acceptor CPE is consistent with EET, there are other mechanisms, such as excited-state electron transfer, which could also give rise to donor PL quenching. To establish whether we indeed observed EET between PFNG*X* and PTAK, we measured the steady-state PL excitation (PLE) spectrum for each CPEC. In this experiment, the PL intensity was measured at a fixed emission wavelength corresponding to emission by the acceptor CPE alone. The excitation wavelength was then varied across the full absorption spectrum of the CPEC while detecting only acceptor PL at the fixed PTAK emission wavelength.

**Figure 7:**
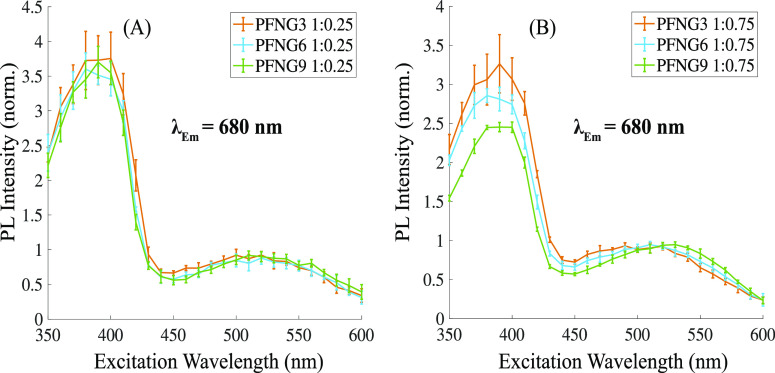
(A) PLE spectra of PFNG*X* CPECs formed with PTAK at a molar charge percentage relative to PFNGX of 25% and normalized to the PTAK peak. (B) Normalized PLE spectra of PFNG*X* CPECs formed with PTAK at a molar charge percentagee of 75%. The emission wavelength was set to 680 nm to ensure that only emission due to PTAK was collected. Error bars correspond to one standard deviation.

CPEC PLE spectra of PFNG3, PFNG6, and PFNG9 each complexed with PTAK at a PFNG*X*/PTAK ionic molar charge ratio of 1:0.25 are shown in Figure 7A, with the emission wavelength fixed at 680 nm. PLE data for the 1:0.5 charge ratio complexes are shown in Figure S31 of the Supporting Information. As shown in Figure 2B, the PFNG*X* chemical series emits negligibly at 680 nm; thus, emission at this wavelength is overwhelmingly due to PTAK. Figure 7A shows that the excitation of all three PFNG*X*/PTAK CPECs at wavelengths where strong PFNG*X* absorption occurs (between 350 and 440 nm) ultimately gives rise to radiative relaxation at 680 nm. Spectra were normalized to the PLE peak in the PTAK absorption region at approximately 520 nm. Even though only PTAK emission is monitored, the PLE spectrum is seen to effectively trace the full CPEC absorption spectrum (Figure 7A). Taken together with the observation of strong donor PL quenching, the PLE spectrum is unambiguous evidence that excitons initially created on the PFNGX donor undergo EET to the PTAK acceptor during their excited-state lifetime.

Since the Stern–Volmer plots showed significant departure from linearity at the high end of the PTAK concentration range corresponding to a 75% molar charge ratio, it is interesting to ask whether the PLE spectrum also undergoes changes between 25 and 75% PTAK. PLE spectra of PFNG3, PFNG6, and PFNG9 CPECs at a 75% molar charge ratio are shown in Figure 7B. It is notable that in the linear Stern–Volmer regime (1:0.25 ratio), all three donor CPEs exhibit effectively identical relative EET efficiencies, as judged by the height of the peak at blue excitation wavelengths, corresponding to absorption by the donor PFNGX. However, for the acceptor ratio that falls outside of the linear Stern–Volmer regime (1:0.75 ratio), there is a clear difference in EET among the three PFNG*X* derivatives. The relative contribution from the low-wavelength peak decreases monotonically as a function of oEG sidechain length. Concomitantly, the wavelength region corresponding to direct PTAK excitation undergoes a subtle redshift that is monotonic in the length of the oEG sidechain.

## 4 Discussion

To summarize, we found that steady-state PL spectra and time-resolved PL decays for the three CPEs were quite similar. The vibronic ratios of the PL spectra are consistent with a highly extended backbone. The crude *l*_p_ estimates from SAXS data are similarly consistent with this interpretation. Interestingly, we found that *l*_p_ increased from PFNG3 to PFNG6, which suggests that the oEG length influences the polymer backbone extension in salt-free aqueous solution, consistent with the small redshift in OD. We also found that the slow diffusive mode and the SAXS intensity were qualitatively different for PFNG9 compared to the other two derivatives. We believe that these observations may point towards an increased propensity for self-assembly of PFNG9.

**Figure 8:**
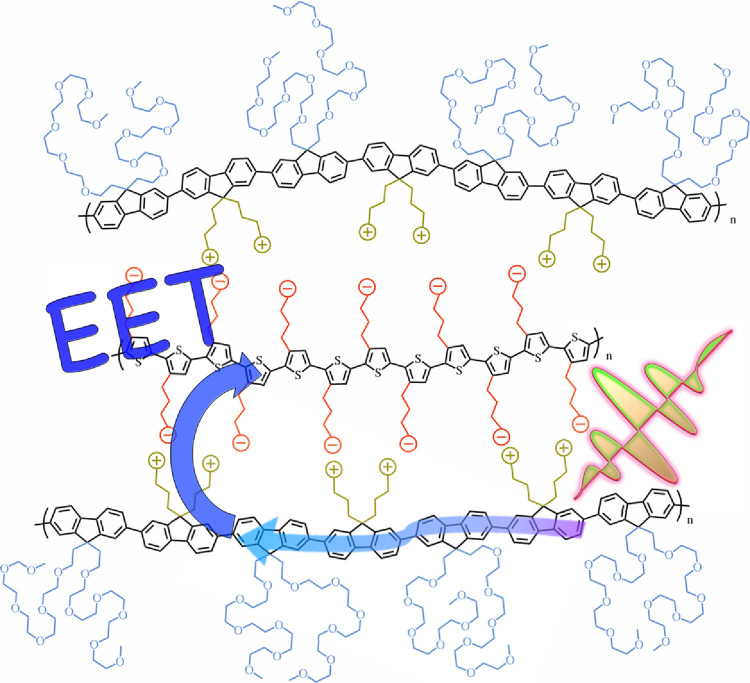
Cartoon of the local inter-CPE complex structure with PFNG6 as the exciton donor and PTAK as the acceptor. Such a simplified structure is likely reflective of complexes at relative ionic CPE stoichiometries well below the 1:1 ratio and at relatively dilute total polymer concentrations. As the macrophase separation point in the vicinity of the 1:1 ratio is approached, we expect the inter-CPE network to evolve toward significantly more complex hierarchical structures.

We originally hypothesized that the EET efficiency between an exciton–donor and an exciton–acceptor CPE would eventually decrease with increasing oEG length. We reasoned that longer oEG chains could hinder complexation between oppositely charged CPEs. It is intriguing that these oEG length differences did not in fact influence the EET efficiency at low acceptor CPE mole ratios as PLE spectra and for all three CPEs were effectively identical. The *K*_SV_ estimates were also fairly similar. This has implications for the determination of the approximate inter-chain structure in the donor–acceptor complex, which is summarized in the cartoon in Figure 8. It must be the case that PFNG*X* polymers for all three oEG lengths arrange in the complex so as to point the oEG sidechains away from the exciton acceptor. Our initial expectation that long oEG sidechains would induce substantial coiling of the exciton donor which would limit complexation was proven incorrect.

Although the relative EET efficiency was independent of oEG length at low acceptor–donor charge mole ratios, at a charge mole ratio of 0.75, intriguing differences in both the Stern–Volmer plots and PLE spectra emerged. First, we found that the β exponent from power law fits to Stern–Volmer curves was largest for PFNG9. Second, we found that the PTAK PLE spectrum for PFNG9 differed from that of PFNG6 in both the decrease in the low-wavelength peak amplitude corresponding to EET from PFNG9 to PTAK, along with the redshift in the long-wavelength (PTAK absorption) region.

Why did the Stern–Volmer plot diverge as [PTAK] was increased? We argue that this divergence corresponds to the onset of spontaneous phase separation, which is expected as the charge mole ratio between the cationic and anionic CPEs approaches unity. It is interesting that, although all three donor CPEs exhibited nearly identical photophysical behavior within CPECs at low acceptor mole fraction, differences emerged near the onset of phase separation. The fact that PFNG9 distinguished itself in these solutions implies that the oEG chains are non-innocent in directing structure formation near the phase transition. At this point, the few-chain complex grows into a large and eventually macroscopic inter-chain network of proximal exciton donors and acceptors, thus forming a highly excitonically interconnected state. Our results suggest that, upon eventual phase separation, we should expect differences in the mechanical and photophysical behavior of dense fluid phases composed of CPECs as a function of oEG sidechain length.^24^

## 5 Conclusions

In this work, we synthesized a chemical series of exciton–donor CPEs with variable lengths of oEG sidechains. The motivation behind this series was to make CPEs containing one ionic and one polar nonionic sidechain for increased stability upon complexation. In salt-free solutions, we found that oEG length influenced the polymer persistence length, at the longest end likely inducing self-assembly of CPE chains. However, when electrostatically coupled with an exciton–acceptor CPE, the EET efficiency was independent of oEG length on the exciton donor, which implies that even very large oEG sidechains do not interfere with efficient exciton transfer within the complex.

Yet, an interesting result is that the oEG length does influence both the apparent onset of phase separation and the likely structure of the inter-CPE complex near this phase transition. The reason why we were interested in increasing the stability of the synthesized exciton–donor CPEs within inter-CPE complexes was due to our desire to form light-harvesting complex fluids. Such fluids can serve as precursors for hierarchical light-harvesting systems via a relatively simple thermodynamic pathway of associative phase separation. The fact that the oEG sidechain evidently influences this associative phase separation has intriguing implications for the tunability of the structure and dynamics within these systems. Our current work is focusing on the phase behavior and exciton transfer within such CPE macrostates in the high-ionic-strength and polymer-concentration limits.

## Supporting Information Available

Details of the synthetic procedures, monomer and polymer characterization, DLS autocorrelation functions, and additional OD, PL, and PLE spectra (PDF)
