# Supplementary material for: Energy Transfer in Aqueous Light Harvesting Antennae Based on Brush-like Inter-Conjugated Polyelectrolyte Complexes
Source: Macromolecules. 2022 Nov 29;55(23):10302–11. doi: 10.1021/acs.macromol.2c01291 (PMC9753961; doi:10.1021/acs.macromol.2c01291)
Supplement: Supplementary file 1 — ma2c01291_si_001.pdf [file ma2c01291_si_001.pdf]

Supporting Information

Energy Transfer in Aqueous Light Harvesting Antennae based on Brush-Like Inter-Conjugated Polyelectrolyte Complexes

Gregory M. Pitch,[1] Levi N. Matsushima,[1] Yannick Kraemer,[1] Eric A. Dailing,[2] Alexander L. Ayzner[1]*

[1] Department of Chemistry and Biochemistry, University of California Santa Cruz, Santa Cruz, CA, 95064, USA

[2] The Molecular Foundry, Lawrence Berkeley National Laboratory, Berkeley, CA, 94720, USA

 Current address: Department of Chemistry, University of California Davis, Davis, CA, 95616, USA

* aayzner@ucsc.edu

S1. Synthesis

1.1. Materials: Reagents and materials were used as received from the following distributors: 2,7-dibromofluorene and 4-toluenesulfonyl chloride were obtained from Oakwood Chemical. Poly[3-(potassium-4-butanoate)thiophene-2,5-diyl] regioregular was obtained from Rieke Metals. Bis(pinacolato)diboron >98% and 1,4-dioxane (anhydrous, 99.8%) were obtained from Alfa Aesar. Triethylene glycol monomethyl ether (>98.0%), hexaethylene glycol monomethyl ether (>96.0%), and nonaethylene glycol monomethyl ether (>93.0%) were obtained from TCI America Inc. Tetraethylammonium bromide 100% was obtained from Chem-Impex International Inc. Palladium catalyst [1,1’-bis(diphenylphosphino)ferrocene]

dichloropalladium (II), dimethyl aminopropyl chloride hydrochloride (96%), sodium hydride (60% dispersed in mineral oil), and dimethylformamide (anhydrous, 99.8%) were obtained from Sigma-Aldrich. Methyl iodide (99.5%), tetrahydrofuran (HPLC grade), and dichloromethane (99.5%) were obtained from Spectrum Chemical. Sodium hydroxide, sodium hydroxide solution (50% w/w), potassium carbonate (anhydrous, 99.7%), triethylamine (99%), diethyl ether (99%), methanol (99.9%), acetone (HPLC grade), chloroform (99.9%), ethyl acetate (99.5%), hexanes (98.5%), and potassium acetate (>99%) were obtained from Fisher Chemical. Dimethyl sulfoxide (99.7%) was obtained from Acros Organics. Chemglass pressure tubes were used for polymerizations and quaternizations (15 mL - part number CG-1880-01). Polyvinylidene difluoride filters (450 nm) were obtained from Lab Safety Supply and polytetrafluoroethylene filters (450 nm) were obtained from Cole-Parmer. $CDCl_3$ (D 99.8%) and D_2O (D 99.9%) were both purchased from Cambridge Isotope Laboratories. Nuclear magnetic resonance (*NMR*) spectra of the monomers were collected on a Bruker Avance III HD 4 channel 500 MHz NMR, and spectra of the polymers were collected on a Bruker Avance III HD 4 channel 800 MHz NMR with a cryoprobe.

S2. Preparation of Reagents

2.1. Reagent TG3: Synthesis of (1-(p-tosyl)-3,6,9- trioxodecane) To a heat gun dried 25 mL round bottom flask in an ice bath, a Teflon coated stir bar, sodium hydroxide (NaOH, 683 mg, 17.1 mmol), deionized water (DI H2O, 3.5 mL, 193.9 mmol), triethylene glycol monomethyl ether (G3OH, 1.00 g, 6.1 mmol), and tetrahydrofuran (THF, 7.0 mL, 86.3 mmol) were added and allowed to react for 30 minutes. Next, p-tolyl sulfonyl chloride (PTSC, 2.11 g,

11.1 mmol) was added under an atmosphere of nitrogen (N2(g)) and the solution was stirred for 12 hours. Upon completion, the reaction contents were poured over 8.7 mL of cold DI H2O, followed by extraction of the product with dichloromethane (DCM, 2 x 4.5 mL). The organic layer was washed with DI H2O (2 x 4.5 mL), brine (1 x 4.5 mL), and subsequently dried over sodium sulfate (Na2SO4). The anhydrous organic layer was decanted away from the drying agent and concentrated under reduced pressure to provide the product TG3 as a colorless oil (77 yield, 1.50 g).2 Figure S1: TG3 1H NMR (500 MHz, CDCl3): *δ* 7.79-7.77 (d, 2H), 7.33-7.31 (d, 2H), 4.14 (t, 2H), 3.67 (t, 2H), 3.59 (m, 2H), 3.57 (s, 4H), 3.51 (t, 2H), 3.35 (s, 3H), 2.43 (s, 3H) Figure S2: TG3 13C NMR (126 MHz, CDCl3): *δ* 144.8, 133.1, 129.8, 128.0, 71.9, 70.7, 70.6, 69.3, 68.7, 59.0, 21.6.

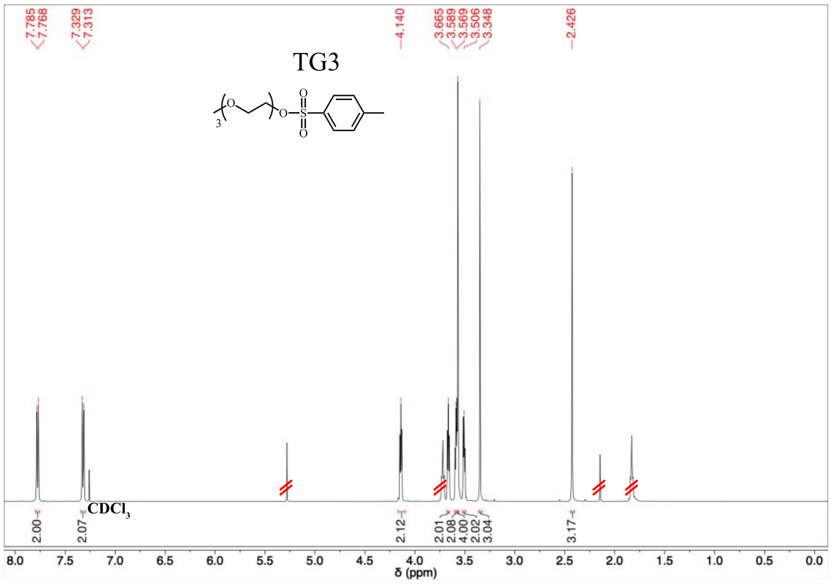

Figure S1: TG3 ^{1}H NMR with trace amount of *DCM, THF,* and Acetone.

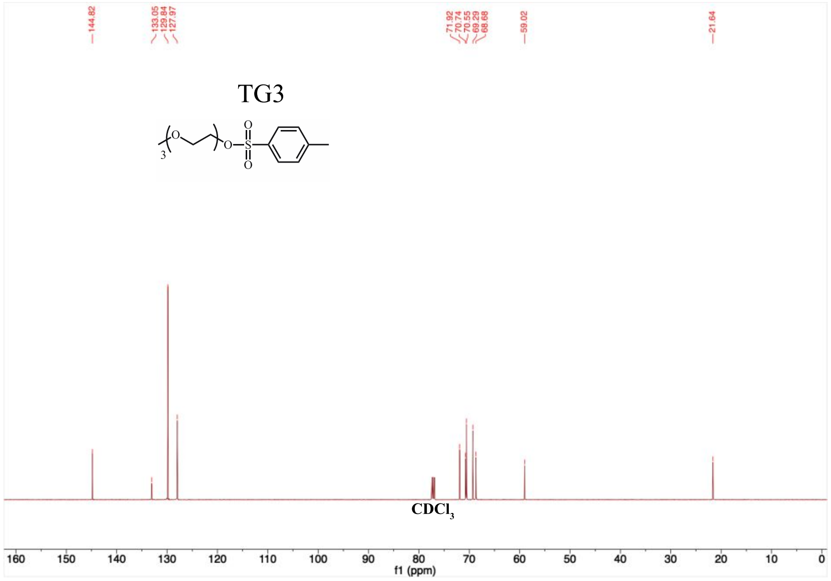

Figure S2: TG3 ^{13}C NMR

2.2. Reagent TG6: Synthesis of (1-(p-tosyl)-3,6,9,12,15,18- hexaoxononadecane) To a heat gun and dried 25 mL round bottom flask in an ice bath, a Teflon coated stir bar, NaOH (1.2 g, 30.0 mmol), DI H2O (6.0 mL, 332.4 mmol), hexaethylene glycol monomethyl ether (G6OH, 3.00 g, 10.3 mmol), and THF (12.0 mL, 148.0 mmol) were added and allowed to react for 30 minutes. Next, PTSC (3.47 g, 18.2 mmol) was added under an atmosphere of N2(g) and reacted for 12 hours. Upon completion, the reaction contents were poured over 16 mL of cold DI H2O, followed by extraction of the product with DCM (4 x 10 mL). The organic layer was washed with DI H2O (2 x 10 mL), brine (1 x 10 mL), and subsequently dried over Na2SO4. The

anhydrous organic layer was decanted away from the drying agent and concentrated under reduced pressure to provide the product TG6 as a colorless oil (99% yield, 4.56 g).2 Figure S3: TG6 1H NMR (500 MHz, CDCl3): δ 7.80-7.79 (d, 2H), 7.35-7.33 (d, 2H), 4.16 (t, 2H), 3.68 (t, 2H), 3.63 (m, 14H), 3.58 (s, 4H), 3.54 (t, 2H), 3.37 (s, 3H), 2.44 (s, 3H) Figure S4: TG6 13C NMR (126 MHz, CDCl3): δ 144.9, 133.2, 130.0, 128.1, 72.1, 70.9, 70.8, 70.7, 70.7, 70.7, 70.7, 69.4, 68.8, 68.1, 59.2, 25.8, 21.8.

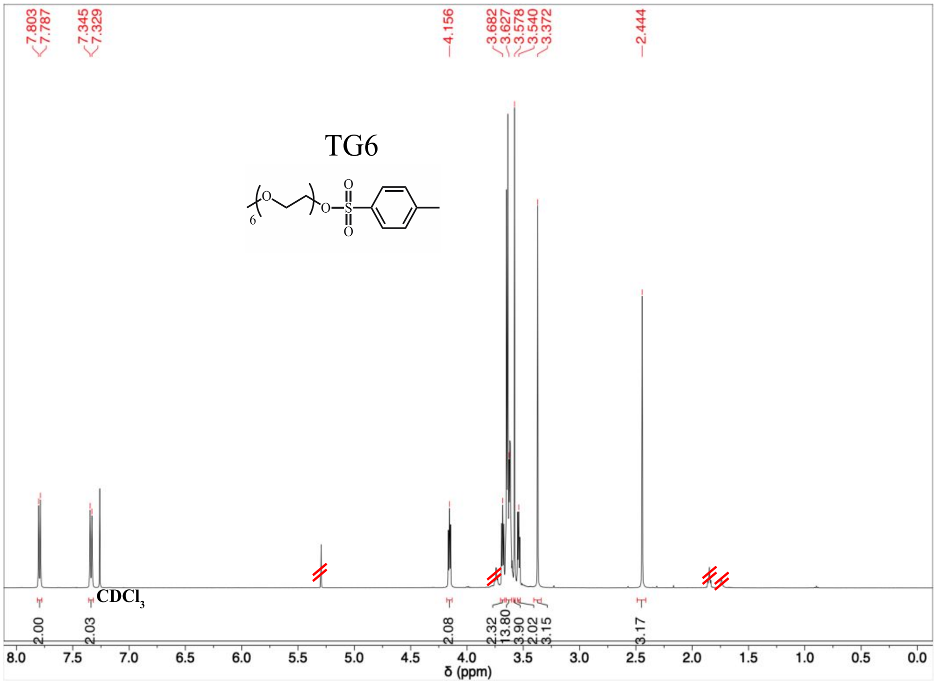

Figure S3: TG6 ^{1}H NMR with trace amount of *DCM, THF,* and water.

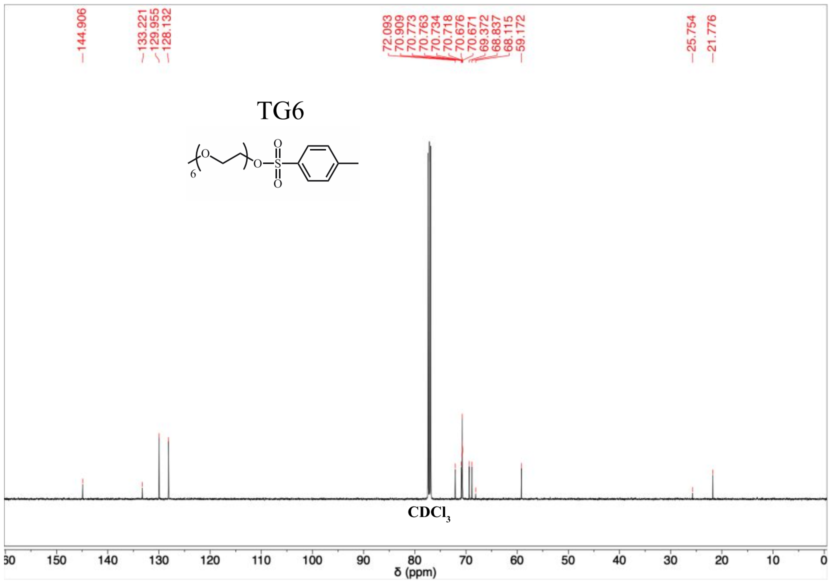

Figure S4: TG6 ^{13}C NMR

2.3. Reagent TG9: Synthesis of (1-(p-tosyl)-3,6,9,12,15,18,21,24,27-nonaoxooctacosane To a heat gun dried 25 mL round bottom flask in an ice bath, a Teflon coated stir bar, NaOH (0.80 g, 20.0 mmol), DI H_2O (4.0 mL, 222 mmol), nonaethylene glycol monomethyl ether (*G9OH*, 3.00 g, 7.0 mmol), and THF (8.0 mL, 98.6 mmol) were added and allowed to react for 30 minutes. Next, PTSC (2.40 g, 12.6 mmol) was added under an atmosphere of N_2(g) and reacted for 12 hours. Upon completion, the reaction contents were poured over 16 mL of cold DI H_2O, followed by extraction of the product with DCM (4 x 10 mL). The organic layer was washed with DI H_2O (2 x 10 mL), brine (1 x 10 mL), and subsequently dried over Na_2SO_4. The anhydrous organic layer was decanted away from the drying agent and concentrated under reduced pressure to provide the product TG9 as a colorless oil (99% yield, 4.06 g).[2] **Figure S5**: TG9 ^1H NMR (500 MHz, $CDCl_3$): δ 7.80-7.79 (d, 2H), 7.35-

7.33 (d, 2H), 4.16 (t, 2H), 3.68 (t, 2H), 3.65- 3.64 (m, 22H), 3.61 (m, 4H), 3.58 (s, 4H), 3.54 (s, 2H), 3.37 (s, 3H), 2.44 (s, 3H) **Figure S6**: TG9 ^{13}C NMR (126 MHz, $CDCl_3$): δ 144.9, 133.2, 130.0, 128.1, 72.1, 70.9, 70.8, 70.7, 70.7, 69.4, 68.8, 68.1, 59.2, 25.8, 21.8.

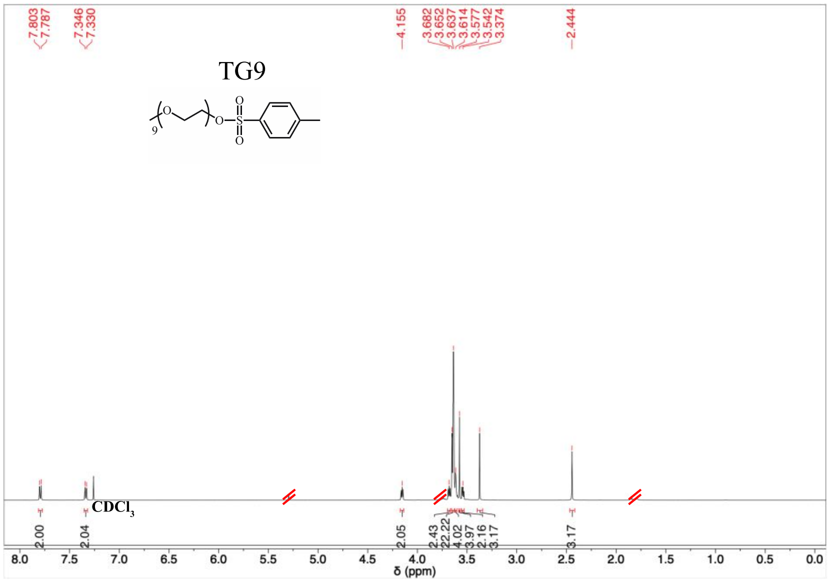

Figure S5: TG9 ^{1}H NMR with trace amount of *DCM, THF,* and water.

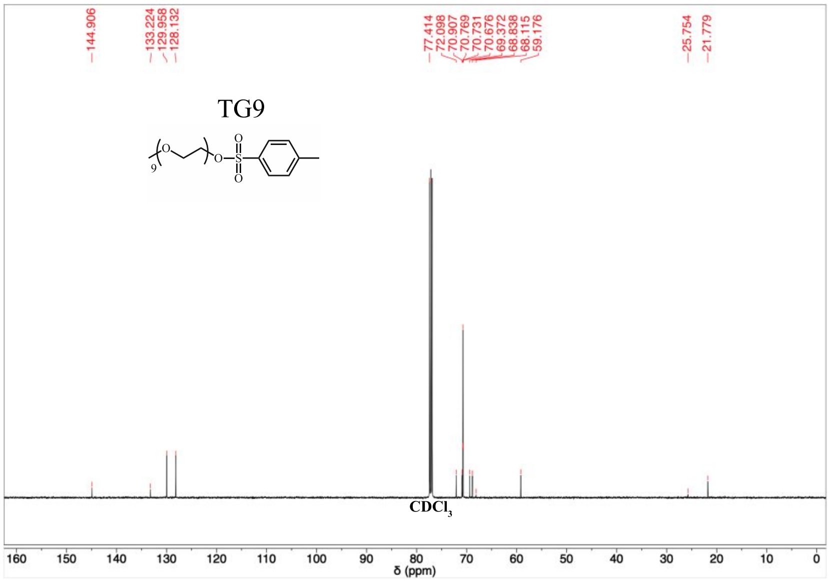

Figure S6: TG9 ^{13}C NMR

2.4. Monomer FG3: Synthesis of (2,7-dibromo-9,9-bis-(2-(2-(2-methoxy-ethoxy)

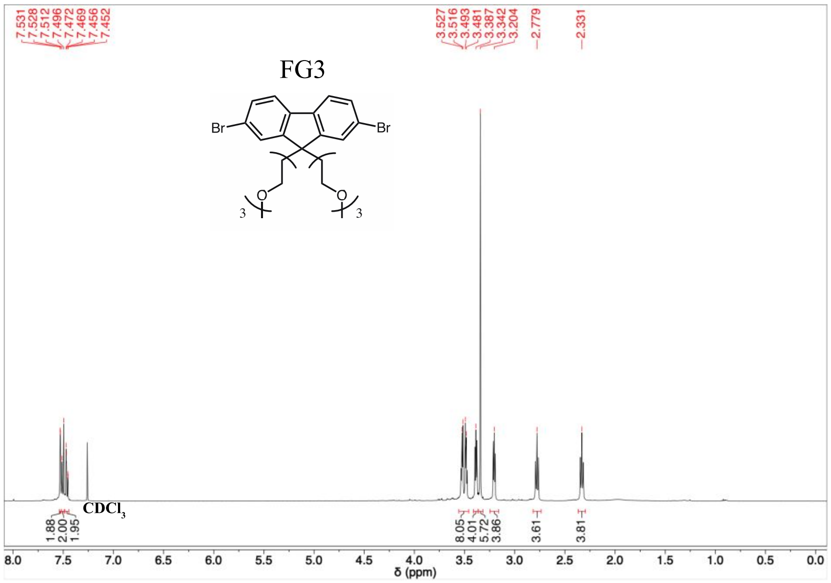

Figure S7: FG3 ^{1}H NMR

ethoxy) ethyl)- fluorene) To a clean and dried 25 mL round bottom flask, a Teflon coated stir

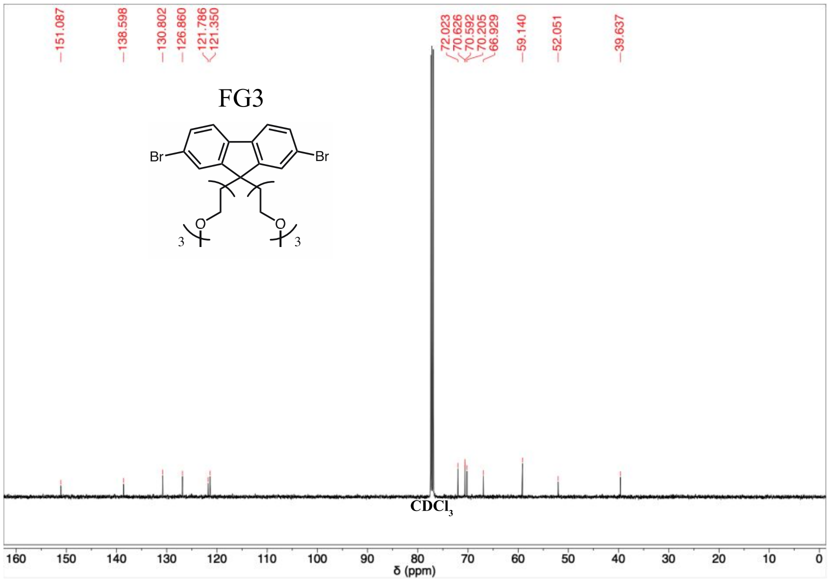

Figure S8: FG3 ^{13}C NMR

bar, F (204.0 mg, 0.63 mmol), anhydrous DMF (3 mL, 38.7 mmol), and sodium hydride (*60% w/w dispersed in mineral oil NaH*, 80 mg, 2.0 mmol) were added under an inert atmosphere of N_2(g). After 30 minutes, the bright red solution was allowed to react at 60 °C for 12 hours with previously prepared TG3 (500 mg, 1.6 mmol). After quenching the remaining NaH with DI H_2O (2 mL), the reaction was extracted with DCM (4 x 20 mL). Dried the combined organic layer over Na_2SO_4. The anhydrous organic layer was decanted away from the drying agent. While stirring, a mixture consisting of 95% DCM with 5% Methanol (*MeOH*) was used to wash the product from the Na_2SO_4 slurry. The DCM: MeOH solution was decanted from the Na_2SO_4 and combined with the organic layer. The organic layer was concentrated under reduced pressure to provide a semi-crude solid which was purified further via silica gel column chromatography (DCM: Ethyl Acetate, 80:20) to obtain FG3 (68.7% yield, 266.8 mg).[3] **Figure S7**: FG3 ^1H NMR (500 MHz, $CDCl_3$): δ 7.53 (d, 2H), 7.51-7.50 (d, 2H), 7.47-7.45 (d, 2H), 3.51 (m, 8H), 3.39 (t, 4H), 3.34 (s, 6H), 3.20 (t, 4H), 2.78 (t, 4H), 2.33 (t, 4H) **Figure S8**: FG3 ^{13}C NMR (126 MHz, $CDCl_3$): δ 151.1, 138.6, 130.8, 126.9, 121.8, 121.4, 72.0, 70.6, 70.6, 70.2, 66.9, 59.1, 52.1, 39.6.

2.5. Monomer FG6: Synthesis of (2,7-dibromo-9,9-bis-(2-(2-(2-(2-(2-methoxy-

ethoxy) ethoxy) ethoxy) ethoxy) ethoxy) ethyl)- fluorene) To a clean and dried 50 mL round

bottom flask, a Teflon coated stir bar, F (870 mg, 2.69 mmol), anhydrous DMF (15 mL, 193.7

mmol), and NaH in 60% w/w dispersed in mineral oil (400 mg, 10.0 mmol) were added under an

inert atmosphere of N_2(g). After 30 minutes, the bright red solution was allowed to react at 60 °C

for 12 hours with previously prepared TG6 (3.0 g, 6.7 mmol). After quenching the remaining

NaH with DI H_2O (20 mL), the reaction was extracted with DCM (4 x 50 mL). Dried the

combined organic layer over Na_2SO_4. The anhydrous organic layer was decanted away from the

drying agent. While stirring, a mixture consisting of 95% DCM with 5% MeOH was used to

wash the product from the Na_2SO_4 slurry. The DCM: MeOH solution was decanted from the Na_2SO_4 and combined with the organic layer. The organic layer was concentrated under reduced pressure to provide a semi-crude solid which was purified further via a silica gel column (Ethyl Acetate: MeOH, 99:1), followed by (Ethyl Acetate: MeOH, 90:10). Since the percentage of MeOH in the solvent used to elute the aggregated fraction of FG6 was 10%, FG6 was dissolved in chloroform (*$CHCl_3$*) to help precipitate out the once dissolved silica gel. The $CHCl_3$ solution was then filtered to remove the precipitate, and concentrated under reduced pressure to obtain FG6 (52.4% yield, 1.23 g).[3] **Figure S9**: FG6 ^{1}H NMR (500 MHz, $CDCl_3$): δ 7.53 (d, 2H), 7.52-7.50 (d, 2H), 7.47-7.46 (d, 2H), 3.64 (m, 12H), 3.61 (m, 8H), 3.58 (m, 4H), 3.54 (t, 8H), 3.38 (m,

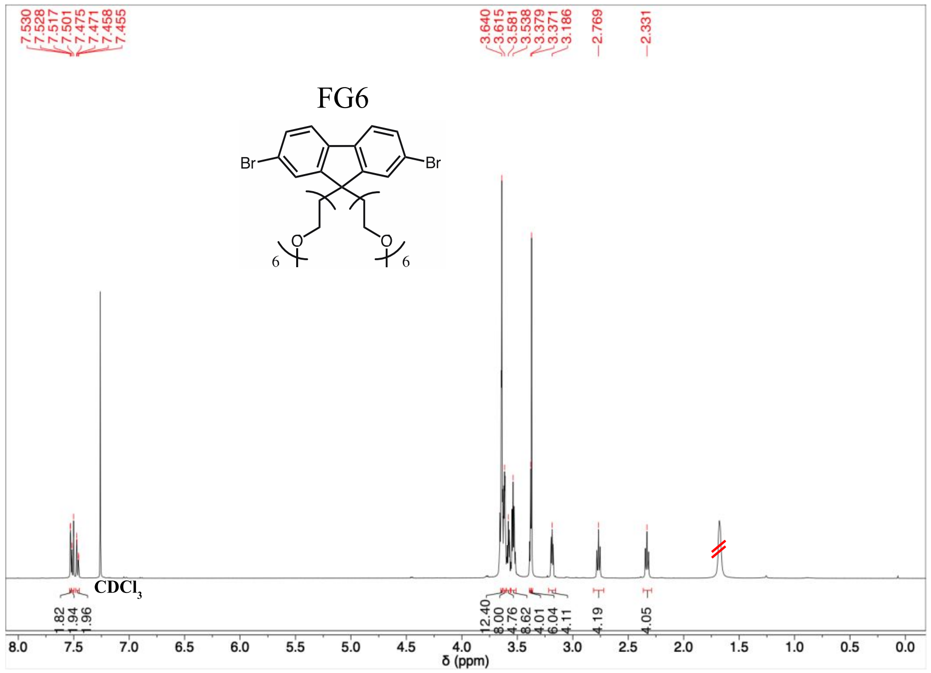

Figure S9: FG6 ^{1}H NMR with trace amount of water.

4H), 3.37 (m, 6H), 3.19 (t, 4H), 2.77 (t, 4H), 2.33 (t, 4H) **Figure S10**: FG6 ^{13}C NMR (126 MHz, $CDCl_3$): δ 151.0, 138.6, 130.8, 126.8, 121.7, 121.3, 72.0, 70.7, 70.6, 70.5, 70.2, 66.9, 59.1, 52.0, 39.6.

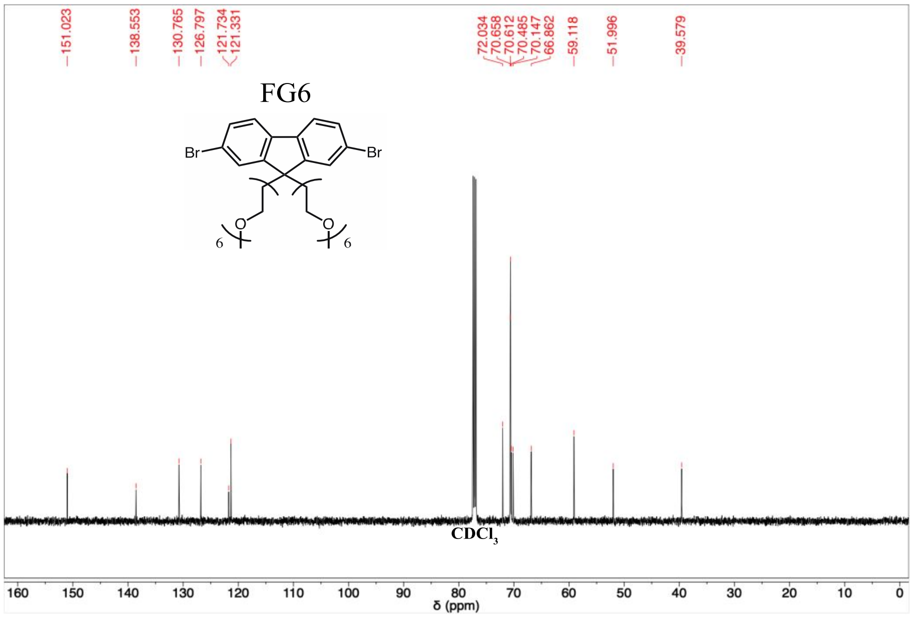

Figure S10: FG6 ^{13}C NMR

2.6. Monomer FG9: Synthesis of (2,7-dibromo-9,9-bis-(2-(2-(2-(2-(2-(2-(2-(2-(2-

methoxy-ethoxy) ethoxy) ethoxy) ethoxy) ethoxy) ethoxy) ethoxy) ethoxy) ethyl)- fluorene) To a

clean and dried 50 mL round bottom flask, a Teflon coated stir bar, F (670 mg, 2.1 mmol),

anhydrous DMF (12 mL, 155 mmol), and NaH in 60% w/w dispersed in mineral oil (210 mg, 5.3

mmol) were added under an inert atmosphere of N_2(g). After 30 minutes, the bright red solution

was allowed to react at 60 °C for 12 hours with previously prepared TG9 (3.0 g, 5.2 mmol).

After quenching the remaining NaH with DI H_2O (15 mL), the reaction was extracted with DCM

(4 x 50 mL). Dried the combined organic layer over Na_2SO_4. The anhydrous organic layer was

decanted away from the drying agent. While stirring, a mixture consisting of 95% DCM with 5%

MeOH was used to wash the product from the Na_2SO_4 slurry. The DCM: MeOH solution was decanted from the Na_2SO_4 and combined with the organic layer. The organic layer was

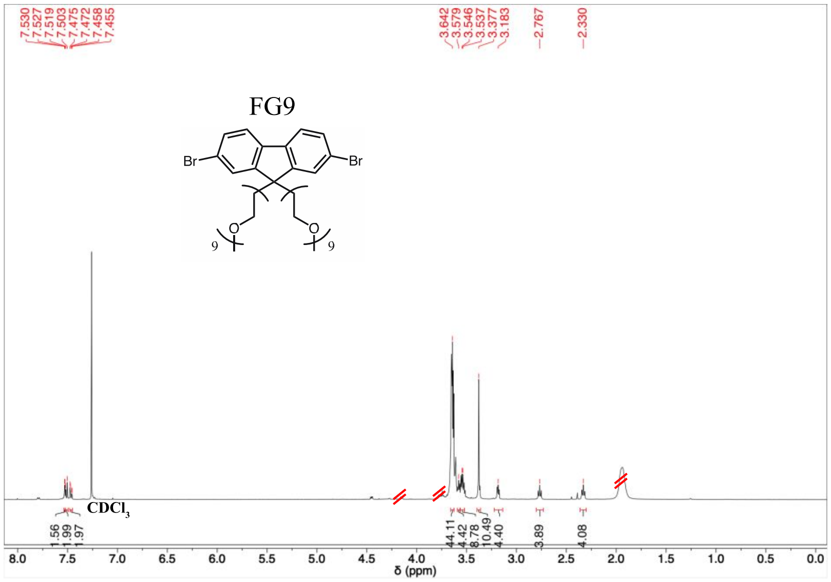

Figure S11: FG9 ^{1}H NMR with trace amount of THF and EtOAc.

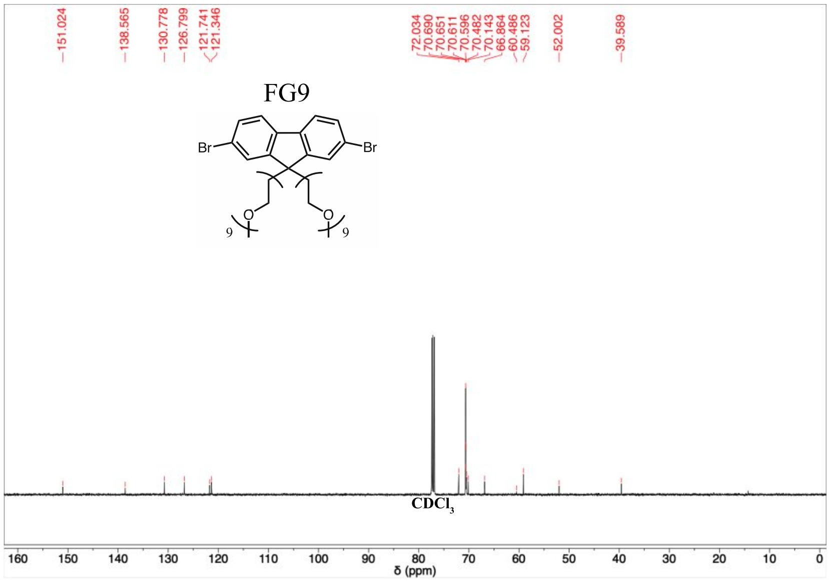

Figure S12: FG9 ^{13}C NMR

concentrated under reduced pressure to provide a semi-crude solid which was purified further via a silica gel column (Ethyl Acetate: MeOH, 90:10). Since the percentage of MeOH in the solvent

used to elute the aggregated fraction of FG9 was 10%, FG9 was dissolved in $CHCl_3$ to help precipitate out the once dissolved silica gel. The $CHCl_3$ solution was then filtered to remove the precipitate, and concentrated under reduced pressure to obtain FG9 (30.0% yield, 0.710 g).[3]
Figure S11: FG9 ^{1}H NMR (500 MHz, $CDCl_3$): δ 7.53 (d, 2H), 7.52-7.50 (d, 2H), 7.47-7.46 (d, 2H), 3.64 (m, 44H), 3.58 (m, 4H), 3.54 (t, 8H), 3.37 (m, 10H), 3.18 (t, 4H), 2.77 (t, 4H), 2.33 (t, 4H) **Figure S12**: FG9 ^{13}C NMR (126 MHz, $CDCl_3$): δ 151.0, 138.6, 130.8, 126.8, 121.7, 121.4, 72.0, 70.7, 70.7, 70.6, 70.6, 70.5, 70.1, 66.9, 60.5, 59.1, 52.0, 39.6.

2.7. Monomer FN: Synthesis of (2,7-dibromo-9,9-bis(3'-(N,N-dimethyl-amino)-propyl)-fluorene) To a clean, dried, 100 mL two neck round bottom flask, a Teflon coated stir bar, dimethyl sulfoxide (*DMSO*, 30.9 mL, 434.6 mmol), 2,7- dibromofluorene (*F*, 2 g, 6.2 mmol), tetrabutylammonium bromide (*TBAB*, 39.8 mg, 0.12 mmol), and 4 mL of a 50 wt. % aqueous sodium hydroxide solution (*50 wt. % aq. NaOH*, 4 mL, 154.3 mmol) was added under an atmosphere of N_2(g). An additional aliquot of DMSO (10.5 mL, 145.1 mmol) was added to the reaction flask, followed by dimethyl aminopropyl chloride hydrochloride salt (*DAPCl*, 2.6 g, 16.4 mmol). The reaction was stirred and heated at 60 °C for 12 hours. Reaction progress was monitored by thin-layer chromatography (*TLC*). DI H_2O (40 mL, 2.216 mmol) was added to the reaction flask to dissolve precipitated salts as well as to solvate DMSO. The product (FN) was extracted from the wet DMSO layer with diethyl ether (*Et_2O*, 8 x 25 mL), washed with a 10 wt. % aqueous NaOH (2 x 50 mL). The organic layer was washed with DI H_2O (3 x 50 mL), followed by a brine wash (1 x 50 mL), and then dried over Na_2SO4. Concentration of the anhydrous organic layer under reduced pressure lead to crude solid which was purified with a silica gel column (Hexanes: Ethyl Acetate: Triethylamine, 49:49:2) to obtain FN (59% yield,

1.81 g).[4,5] **Figure S13**: FN ^{1}H NMR (500 MHz, $CDCl_3$): δ 7.52-7.50 (d, 2H), 7.47 (s, 2H), 7.45-7.44 (d, 2H), 2.03 (s, 12H), 1.99 (m, 8H), 0.77 (m, 4H) **Figure S14**: FN ^{13}C NMR (126 MHz,

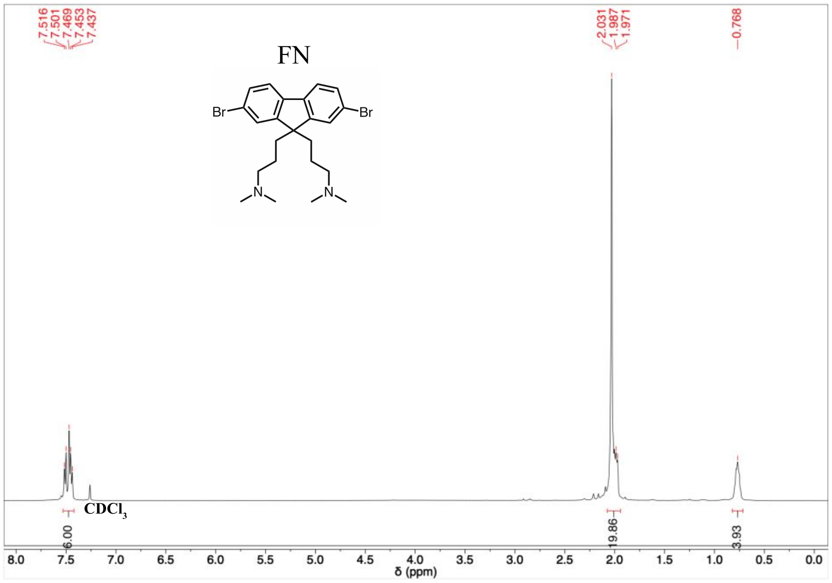

Figure S13: FN ^{1}H NMR

$CDCl_3$): δ 152.2, 139.3, 130.6, 126.3, 121.8, 121.4, 59.7, 55.5, 45.5, 37.7, 22.2.

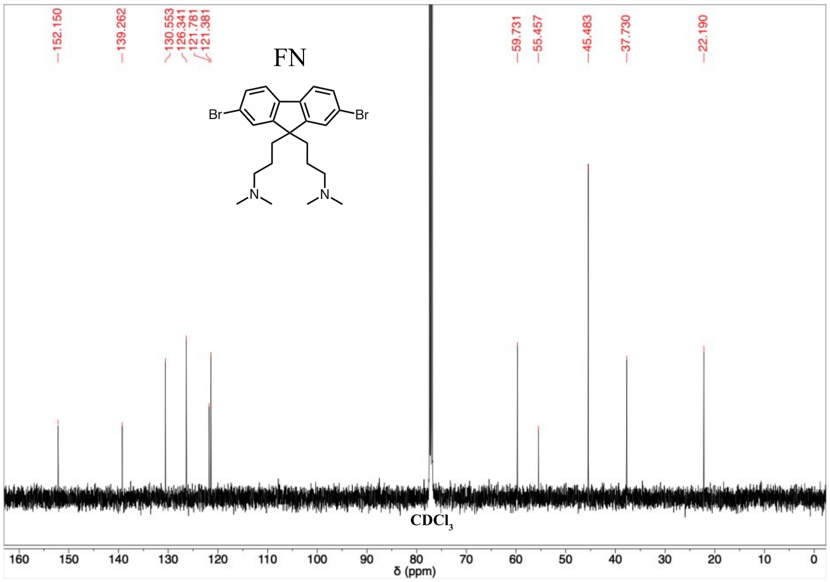

Figure S14: FN ^{13}C NMR

2.8. Monomer FNB: Synthesis of (2,7-diboryl pinacol ester-9,9-bis(3'-(N,N-dimethyl-amino)-propyl)-fluorene) To a clean, dried, 100 mL two neck round bottom flask, a Teflon coated stir bar, dimethylformamide (*DMF*, 39 mL, 505.8 mmol), FN (1 g, 2.0 mmol), bis(pinacolato)diboron (*B_2Pin_2*, 2.26 g, 8.9 mmol), potassium acetate (*KOAc*, 3.53 g, 17.8 mmol), [1,1'-Bis(diphenyl-phosphino)ferrocene]dichloro-palladium(II) (*$Pd(dppf)Cl_2$*, 0.296 g, 0.40 mmol) were added under an atmosphere of N_2(g). The contents of the reaction were stirred and heated at 80 °C for 24 hours. Reaction progress was monitored by TLC. Upon completion, the reaction was concentrated to dryness, and the crude solid was extracted with hot HPLC-grade hexanes (7 x 100 mL). The combined hexanes layer was filtered, concentrated to dryness, reextracted with hot hexanes, and re-concentrated to dryness. Acetone was used to extract the product from the re-dried hexanes layer and was allowed to crystallize out of the solution as an off-white solid. The crystals of FNB were collected via filtration and washed with a minimal amount of cold acetone to obtain FNB (55% yield, 0.6647 mg).[4,5] **Figure S15**: FNB ^{1}H NMR (500 MHz, $CDCl_3$): δ 7.80-7.78 (d, 2H), 7.78 (s, 2H), 7.71-7.70 (d, 2H), 2.06 (m, 4H), 1.99 (m, 4H), 1.98 (s, 12H), 1.37 (s, 24H), 0.75 (m, 4H) **Figure S16**: FNB ^{13}C NMR (126 MHz, $CDCl_3$): δ 149.4, 144.0, 134.3, 129.1, 119.8, 84.0, 59.2, 54.7, 44.6, 37.4, 31.1, 25.1.

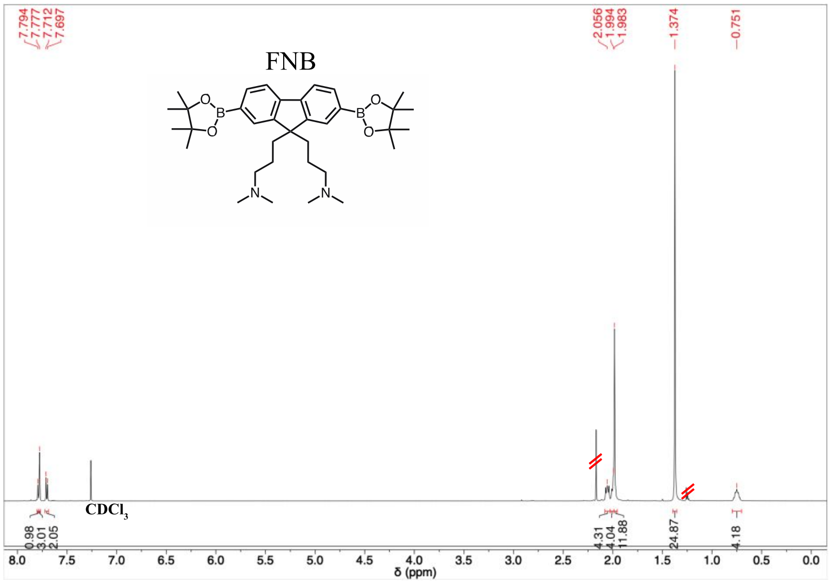

Figure S15: FNB ^{1}H NMR with trace amount of acetone and hexanes.

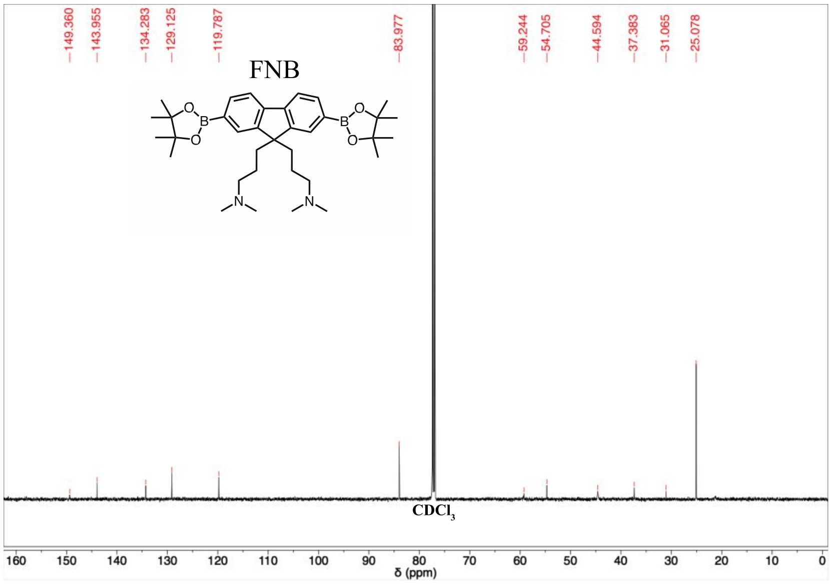

Figure S16: FNB ^{13}C NMR

S3. Synthesis of Neutral Polymers

3.1. Polymer nPFNG3: Polymerization of poly([9,9-bis(3'-(N,N-dimethyl-amino)-propyl)-fluorene]-alt-co-[9,9-bis-(2-(2-(2-methoxy-ethoxy) ethoxy) ethyl)- fluorene] To a clean and dried 15 mL pressure tube, a Teflon coated stir bar, FG3 (500 mg, 0.81 mmol), FNB (480 mg, 0.82 mmol), potassium carbonate (K_2CO_3, 1.35 g, 9.8 mmol), 1,4- dioxane (*Dioxane*, 7 mL, 90.4 mmol), DI H_2O (5.0 mL, 277.0 mmol), and Pd(dppf)Cl_2 (6 mg, 0.008 mmol) were added. The reaction solution as well as the head space was flushed with N_2(g). The pressure vial was capped quickly, placed into a silicon oil bath, stirred, and heated at 100 °C for 48 hours. To stop the reaction, the stirring function was turned off, the bottom water layer was removed, and an aliquot of nPFNG3 dispersed in dioxane was removed for further characterization. The polymer in dioxane (1 mL) was pipetted into DI H_2O (10 mL) to induce precipitation of nPFNG3. The water was decanted from the polymer and nPFNG3 was dried via vacuum filtration.[1,6] **Figure S17**: nPFNG3 ^{1}H NMR (800 MHz, $CDCl_3$): δ 7.82-7.70 (br, 1.00 H), 3.70 (br, 1.11 H), 3.64 (br, 0.12 H), 3.52 (br, 0.35 H), 3.45 (br, 0.85 H), 3.13 (br, 0.91 H), 2.93 (br, 0.26 H), 2.79 (br, 0.04 H), 2.57 (br, 0.19 H), 2.47 (br, 0.09 H), 2.42 (br, 0.14 H), 2.18 (br, 0.12 H), 2.11 (br, 0.32 H), 2.06 (br, 0.98 H), 0.97- 0.89 (br, 0.30 H) **Figure S18**: nPFNG3- ^{13}C NMR (200 MHz, $CDCl_3$): δ 151.5, 150.0, 141.0, 140.5, 139.6, 131.6, 129.1, 128.4, 126.9, 126.6, 121.7, 121.5, 120.4, 72.0, 70.7, 70.6, 69.8, 68.7, 67.2, 66.5, 66.3, 62.8, 62.4, 59.9, 59.1, 55.3, 51.6, 45.5, 40.1, 38.1, 29.8, 22.3.

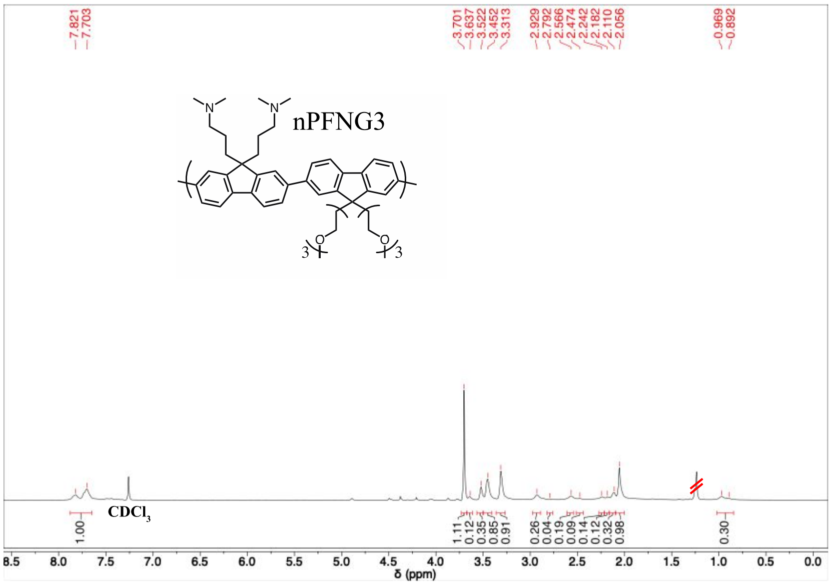

Figure S17: nPFNG3- ^{1}H NMR

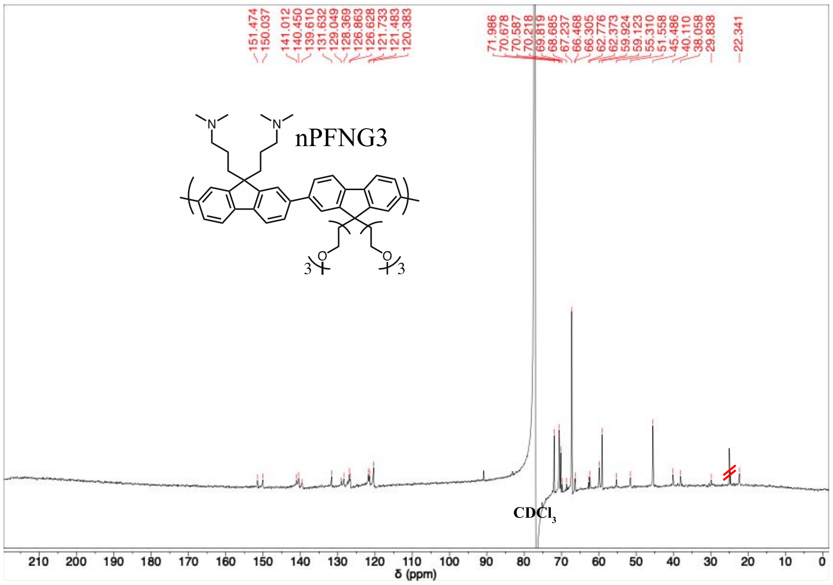

Figure S18: nPFNG3- ^{13}C NMR

3.2. Polymer nPFNG6: <u>Polymerization of nPFNG6: poly([9,9-bis(3'-(N,N-dimethyl-amino)-propyl)-fluorene]-alt-co-[9,9-bis-(2-(2-(2-(2-(2-methoxy-ethoxy) ethoxy) ethoxy) ethoxy) ethoxy) ethyl)- fluorene]:</u> To a clean and dried 15 mL pressure tube, a Teflon coated stir bar, FG6 (500 mg, 0.57 mmol), FNB (344.08 mg, 0.58 mmol), K_2CO_3 (0.950 g, 6.9 mmol),

Dioxane (5 mL, 64.6 mmol), DI H_2O (3.4 mL, 221.6 mmol), and Pd(dppf)Cl_2 (4 mg, 0.005 mmol) were added. The reaction solution as well as the head space was flushed with N_2(g). The pressure vial was capped quickly, placed into a silicon oil bath, stirred, and heated at 100 °C for 48 hours. To stop the reaction, the stirring function was turned off, the bottom water layer was removed, and an aliquot of nPFNG6 dispersed in Dioxane was removed for further characterization. The polymer in Dioxane (1 mL) was pipetted into DI H_2O (10 mL) to induce precipitation of nPFNG6. The water was decanted from the polymer and nPFNG6 was dried via vacuum filtration.[1,6] **Figure S19**: nPFNG6- ^{1}H NMR (800 MHz, $CDCl_3$): δ 7.83-7.71 (br, 1.00 H), 3.62 (br, 2.28 H), 3.52 (br, 1.15 H), 3.44 (br, 0.51 H), 3.35 (br, 0.65 H), 3.28 (br, 0.40 H), 2.91 (br, 0.34 H), 2.56 (br, 0.33 H), 2.18 (br, 0.82 H), 2.10 (br, 0.93 H), 1.00- 0.87 (br, 0.31 H), **Figure S20**: nPFNG6- ^{13}C NMR (200 MHz, $CDCl_3$): δ 151.2, 150.0, 140.9, 140.5, 140.3, 139.5, 126.9, 126.7, 121.78, 121.5, 120.4, 72.1, 70.6, 70.2, 67.3, 67.2, 66.3, 62.4, 59.7, 59.2, 55.2, 54.5, 51.5, 45.2, 44.5, 40.1, 37.9, 22.0, 21.1.

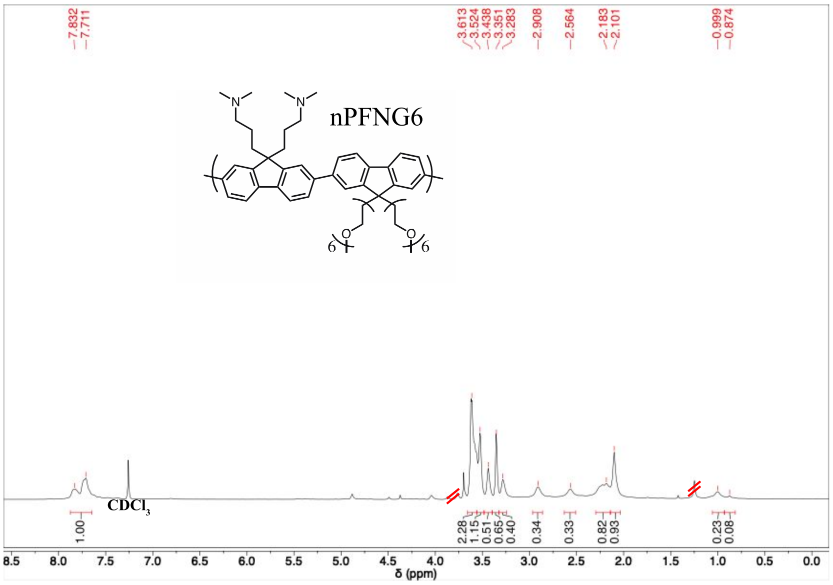

Figure S19: nPFNG6- ^{1}H NMR

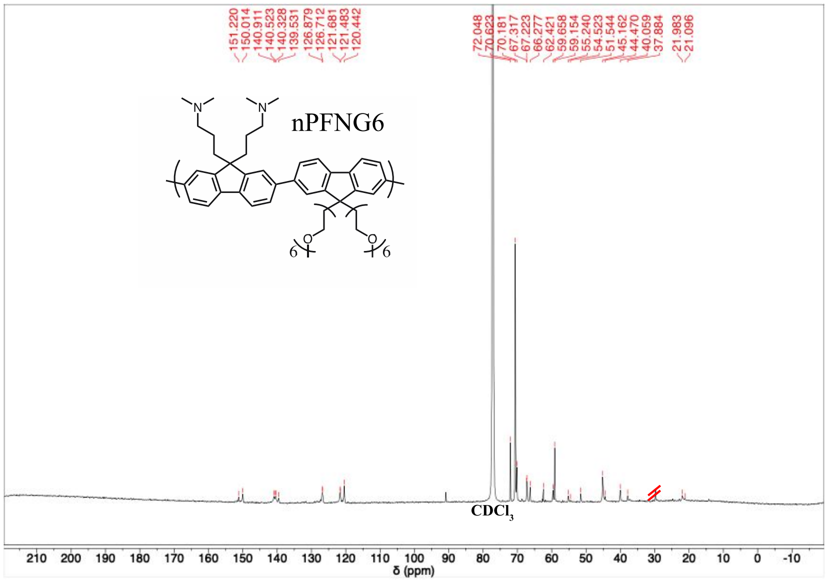

Figure S20: nPFNG6- ^{13}C NMR

3.3. Polymer nPFNG9: Polymerization of poly([9,9-bis(3'-(N,N-dimethyl-amino)-propyl)-fluorene]-alt-co-[9,9-bis-(2-(2-(2-(2-(2-(2-(2-(2-(2-methoxy-ethoxy) ethoxy) ethoxy) ethoxy) ethoxy) ethoxy) ethoxy) ethoxy) ethyl)- fluorene]: To a clean and dried 15 mL pressure tube, a Teflon coated stir bar, FG9 (500 mg, 0.44 mmol), FNB (260 mg, 0.44 mmol), K_2CO_3 (0.780 g, 5.6 mmol), Dioxane (4 mL, 54.4 mmol), DI H_2O (3.0 mL, 166.2 mmol), and $Pd(dppf)Cl_2$ (3.0 mg, 0.004 mmol) were added. The reaction solution as well as the head space was flushed with N_2(g). The pressure vial was capped quickly, placed into a silicon oil bath, stirred, and heated at 100 °C for 48 hours. To stop the reaction, the stirring function was turned off, the bottom water layer was removed, and an aliquot of nPFNG9 dispersed in Dioxane was removed for further characterization. The polymer in Dioxane (1 mL) was pipetted into DI H_2O (10 mL) to induce precipitation of nPFNG9. The water was decanted from the polymer and nPFNG6 was dried via vacuum filtration.[1,6] **Figure S21**: nPFNG9- ^{1}H NMR (800 MHz, $CDCl_3$):

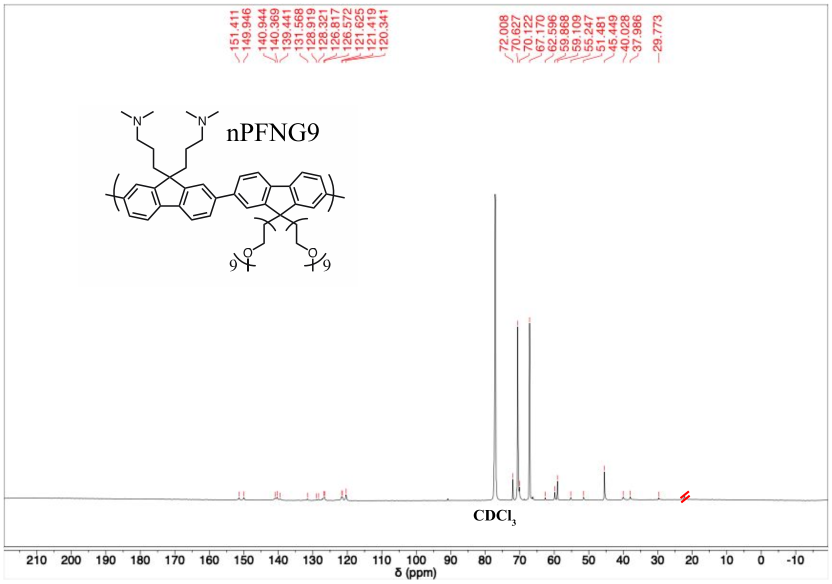

Figure S22: nPFNG9- ^{13}C NMR

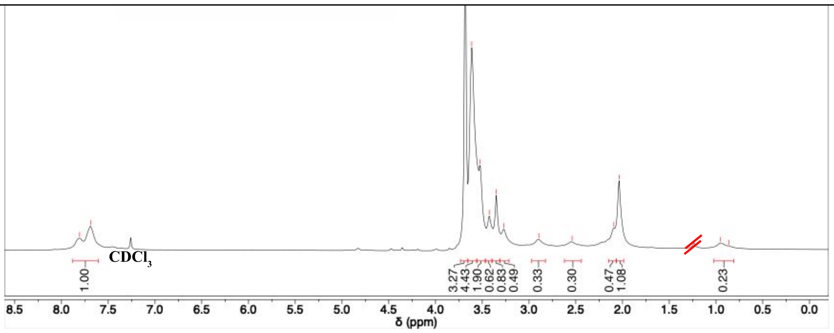

Figure S21: nPFNG9- ^{1}H NMR

δ 7.81- 7.69 (br, 1.00 H), 3.68 (br, 3.27 H), 3.61 (br, 4.43 H), 3.52 (br, 1.90 H), 3.43 (br, 0.62 H), 3.35 (br, 0.83 H), 3.27 (br, 0.49 H), 2.90 (br, 0.33 H), 2.54 (br, 0.30 H), 2.09 (br, 0.47 H), 2.04 (br, 1.08 H), 0.95- 0.86 (br, 0.23 H) **Figure S22**: nPFNG9- ^{13}C NMR (200 MHz, $CDCl_3$): δ 151.4, 150.0, 140.9, 140.4, 139.4, 131.6, 128.9, 128.3, 126.8, 126.6, 121.6, 121.4, 120.3, 72.0, 70.6, 70.1, 67.2, 62.6, 59.9, 59.1, 55.2, 51.5, 45.5, 40.0, 38.0, 29.8.

S4. Synthesis of Conjugated Polyelectrolytes

4.1. Conjugated Polyelectrolyte PFNG3: Conjugated Polyelectrolyte poly([9,9-bis(3'-

(N,N,N-trimethyl-ammonium)-propyl)-fluorene]-alt-co-[9,9-bis-(2-(2-(2-methoxy-ethoxy)

ethoxy) ethyl)- fluorene]: In-situ quaternization of nPFNG3 occurred via the addition of methyl

iodide (MeI, 4.0 mL, 64.3 mmol) directly to the remaining dioxane layer containing nPFNG3.

After reacting for 2 hours, a small aliquot of DI H_2O was added to help dissolve the precipitated polymer. The reaction was heated to 50 °C for 5 days and DI H_2O was added until there was no

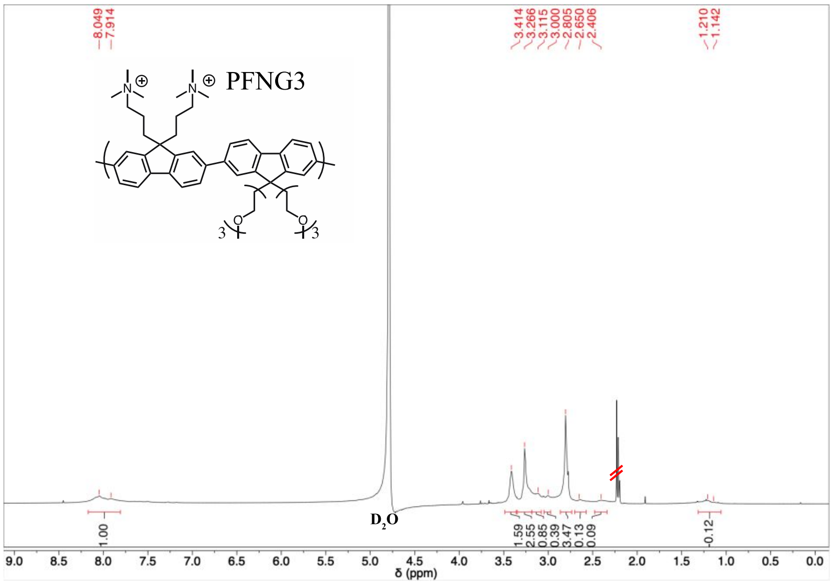

Figure S23: PFNG3- ^{1}H NMR with trace amount of acetone.

more headspace in the pressure vial. After 5 days, the remaining MeI was quenched with a solution of NaOH. Upon addition of NaOH, some of the newly formed PFNG3 precipitated out of the solution. The solution along with some precipitate was dialyzed via Snakeskin dialysis tubing (10,000 MWCO) submerged in a vat of DI H_2O. The DI H_2O was replaced with fresh DI H_2O every day for 5 days. After 5 days, the dialyzed solution of PFNG3 was concentrated under reduced pressure, filtered, transferred into a 50 mL Falcon tube, and lyophilized to yield PFNG3 as a yellow solid (60 mg, 9.4% yield).[1,6] **Figure S23**: PFNG3- ^{1}H NMR (800 MHz, D_2O): δ 8.05- 7.91 (br, 1.00 H), 3.41 (br, 1.59 H), 3.27 (br, 2.55 H), 3.12 (br, 0.85 H), 3.00 (br, 0.39 H), 2.81 (br, 3.47 H), 2.65 (br, 0.13 H), 2.41 (br, 0.09 H), 1.21- 1.14 (br, 0.12 H) **Figure S24**: PFNG3- ^{13}C NMR (200 MHz, D_2O): δ 215.6, 161.1, 150.4, 140.6, 140.0, 127.4, 121.6, 120.9, 70.9, 69.3, 68.9, 67.0, 66.5, 58.0, 54.6, 52.5, 51.9, 38.7, 38.3, 35.3, 30.2, 30.1, 29.96, 29.87, 29.7, 29.6, 29.5, 17.6.

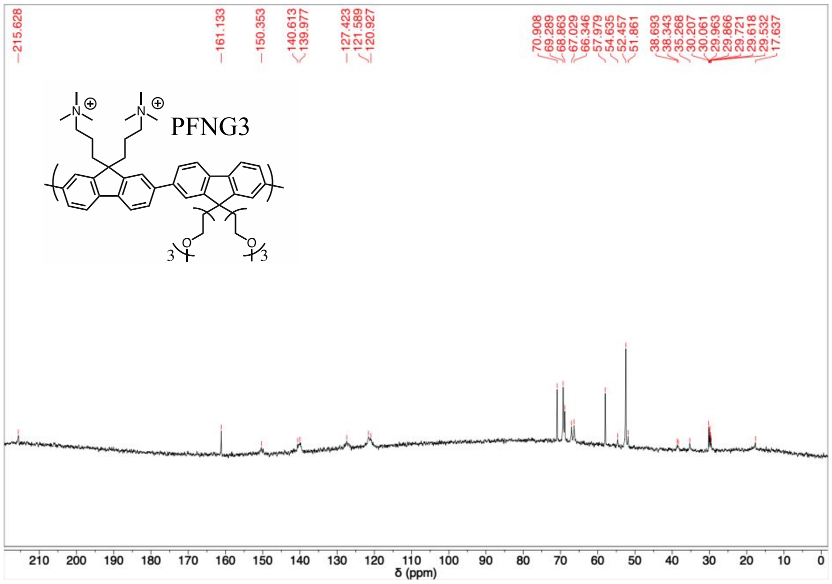

Figure S24: PFNG3- ^{13}C NMR

4.2. Conjugated Polyelectrolyte PFNG6: Conjugated polyelectrolyte poly([9,9-bis(3'-(N,N,N-trimethyl-ammonium)-propyl)-fluorene]-alt-co-[9,9-bis-(2-(2-(2-(2-(2-(2-methoxy-ethoxy) ethoxy) ethoxy) ethoxy) ethoxy) ethyl)- fluorene]: In-situ quaternization of nPFNG6 occurred via the addition of methyl iodide (MeI, 2.5 mL, 40.2 mmol) directly to the remaining dioxane layer containing nPFNG6. After reacting for 2 hours, a small aliquot of DI H_2O was added to help dissolve the precipitated polymer. The reaction was heated to 50 °C for 5 days and DI H_2O was added until there was no more headspace in the pressure vial. After 5 days, the remaining MeI was quenched with a solution of NaOH. Upon addition of NaOH, some of the newly formed PFNG6 precipitated out of the solution. The solution along with some precipitate was dialyzed via Snakeskin dialysis tubing (10,000 MWCO) submerged in a vat of DI H_2O. The

DI H_2O was replaced with fresh DI H_2O every day for 5 days. After 5 days, the dialyzed solution of PFNG6 was concentrated under reduced pressure, filtered, transferred into a 50 mL Falcon tube, and lyophilized to yield PFNG6 as a dark yellow solid (240 mg, 40.1% yield).[1,6] **Figure S25**: PFNG6- ^{1}H NMR (800 MHz, D_2O): δ 8.06- 7.93 (br, 1.00 H), 3.59 (br, 4.79 H), 3.43 (br, 0.84 H), 3.31 (br, 1.55 H), 3.24 (br, 0.74 H), 3.01 (br, 0.64 H), 2.84 (br, 3.25 H), 2.65 (br, 0.43 H), 2.41 (br, 0.20 H), 1.25- 1.16 (br, 0.16 H) **Figure S26**: PFNG6- ^{13}C NMR (800 MHz, D_2O): δ 215.7, 161.3, 150.4, 149.8, 140.6, 140.2, 127.4, 126.8, 121.7, 121.1, 120.9, 71.0, 69.5, 69.0, 67.1, 66.3, 58.0, 54.6, 52.5, 51.9, 38.7, 35.4, 30.2, 30.1, 30.0, 29.9, 29.7, 29.6, 17.8, 17.6.

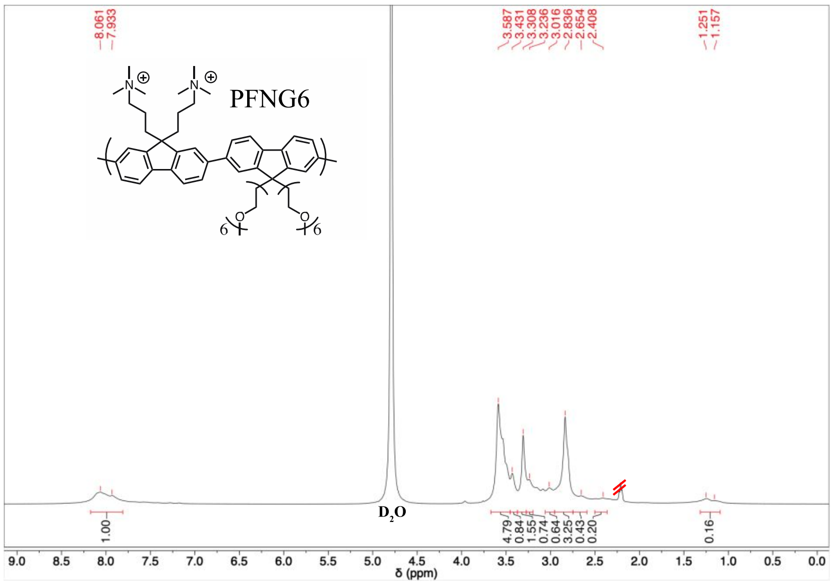

Figure S25: PFNG6- ^{1}H NMR with trace amount of acetone.

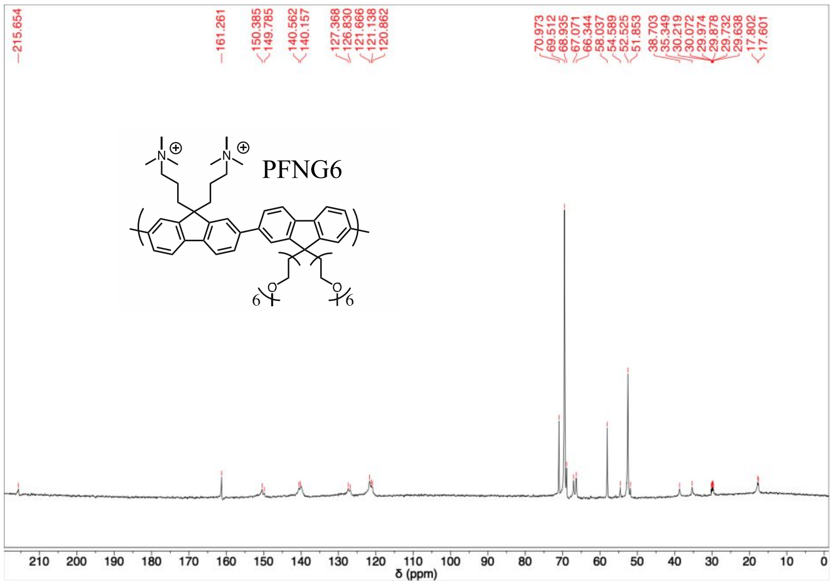

Figure S26: PFNG6- ^{13}C NMR

4.3. Conjugated Polyelectrolyte PFNG9: Conjugated polyelectrolyte poly([9,9-bis(3'-(N,N,N-trimethyl-ammonium)-propyl)-fluorene]-alt-co-[9,9-bis-(2-(2-(2-(2-(2-(2-(2-(2-methoxy-ethoxy) ethoxy) ethoxy) ethoxy) ethoxy) ethoxy) ethoxy) ethoxy) ethyl)- fluorene]: In-situ quaternization of nPFNG9 occurred via the addition of methyl iodide (MeI, 1.5 mL, 24.1 mmol) directly to the remaining dioxane layer containing nPFNG9. After reacting for 2 hours, a small aliquot of DI H_2O was added to help dissolve the precipitated polymer. The reaction was heated to 50 °C for 5 days and DI H_2O was added until there was no more headspace in the pressure vial. After 5 days, the remaining MeI was quenched with a solution of NaOH. Upon addition of NaOH, some of the newly formed PFNG6 precipitated out of the solution. The solution along with some precipitate was dialyzed via Snakeskin dialysis tubing (10,000 MWCO) submerged in a vat of DI H_2O. The DI H_2O was replaced with fresh DI H_2O every day

for 5 days. After 5 days, the dialyzed solution of PFNG9 was concentrated under reduced pressure, filtered, transferred into a 50 mL Falcon tube, and lyophilized to yield PFNG9 as a

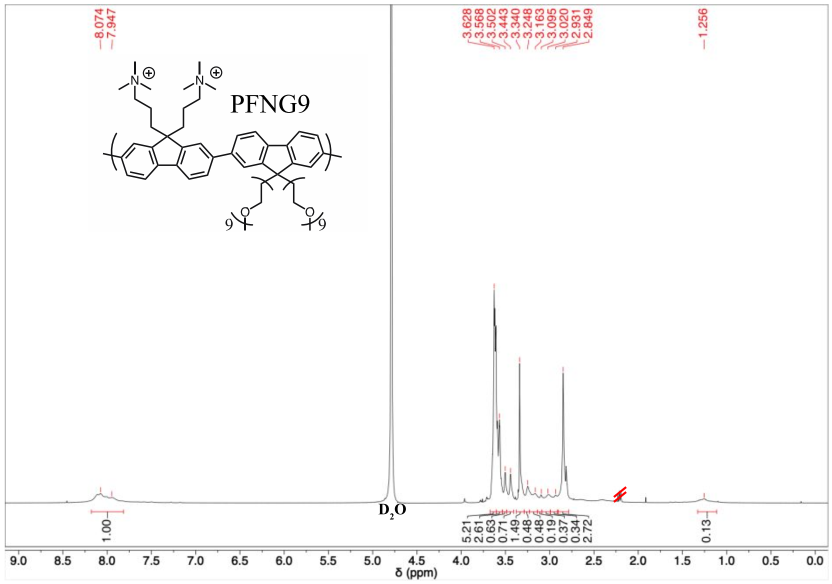

Figure S27: PFNG9- ^{1}H NMR with trace amount of acetone.

brown solid (60 mg, 10.4% yield).[1,6] **Figure S27**: PFNG9- ^{1}H NMR (800 MHz, D_2O): δ 8.07-7.95 (br, 1.00 H), 3.63 (br, 5.21 H), 3.57 (br, 2.61 H), 3.50 (br, 0.63 H), 3.44 (br, 0.71 H), 3.34 (br, 1.49 H), 3.25 (br, 0.48 H), 3.16 (br, 0.48 H), 3.10 (br, 0.19 H), 3.02, (br, 0.37 H), 2.93 (br, 0.34 H), 2.85 (br, 2.72 H), 1.26 (br, 0.13 H) **Figure S28**: PFNG9- ^{13}C NMR (800 MHz, D_2O): δ 215.7, 161.6, 150.4, 149.8, 140.5, 139.8, 127.3, 126.8, 121.6, 120.9, 70.1, 71.0, 69.6, 69.0, 67.1, 66.37, 62.48, 58.1, 54.6, 52.6, 51.8, 38.6, 35.4, 29.8, 17.9.

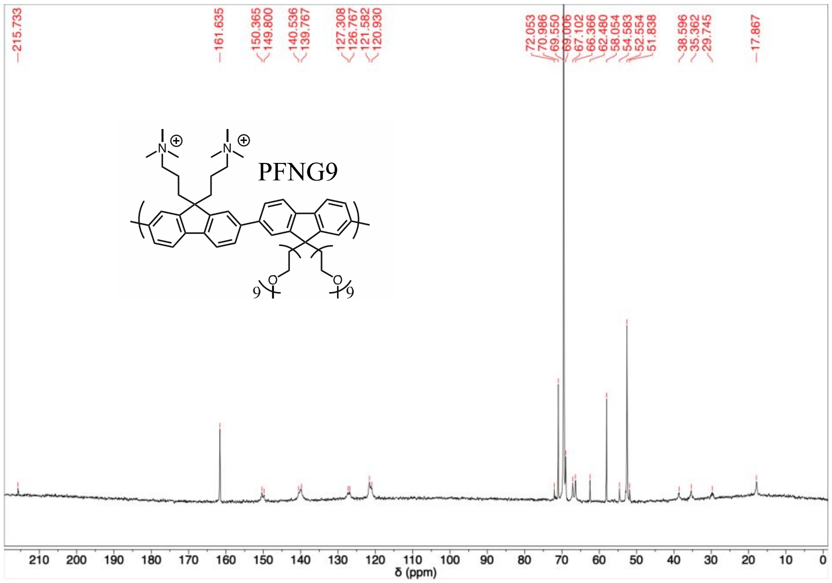

Figure S28: PFNG9- ^{13}C NMR

S5. Dynamic Light Scattering.

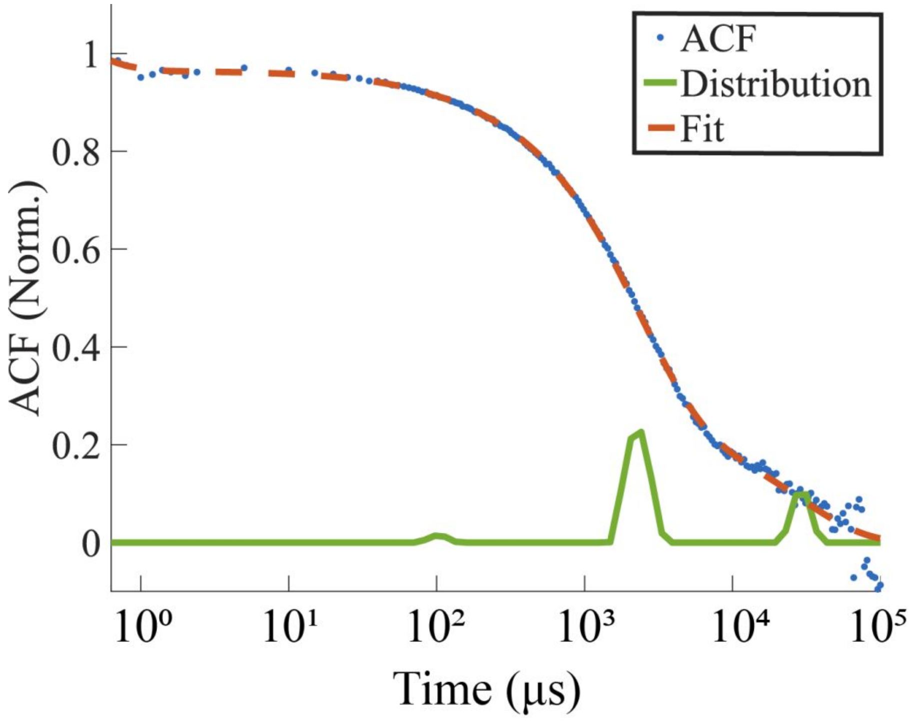

Figure S29: Normalized DLS autocorrelation function (ACF) of an aqueous PFNG9 solution (blue circles). The ACF clearly displays a bimodal distribution of decay times (shown in green). The distribution function was obtained by fitting the ACF using the CONTIN method. The scattering angle was 67º.

S6. Additional Spectroscopic Characterization.

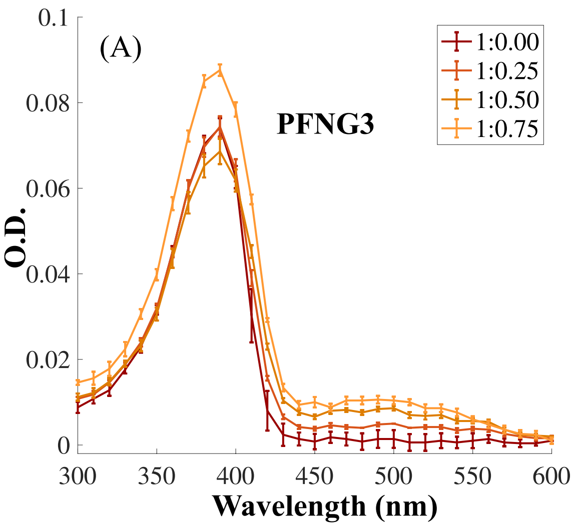

Figure S30: Optical density spectra of (A) PFNG3 and (B) PFNG9 complexes with PTAK as a function of the PFNGX:PTAK molar charge ratio.

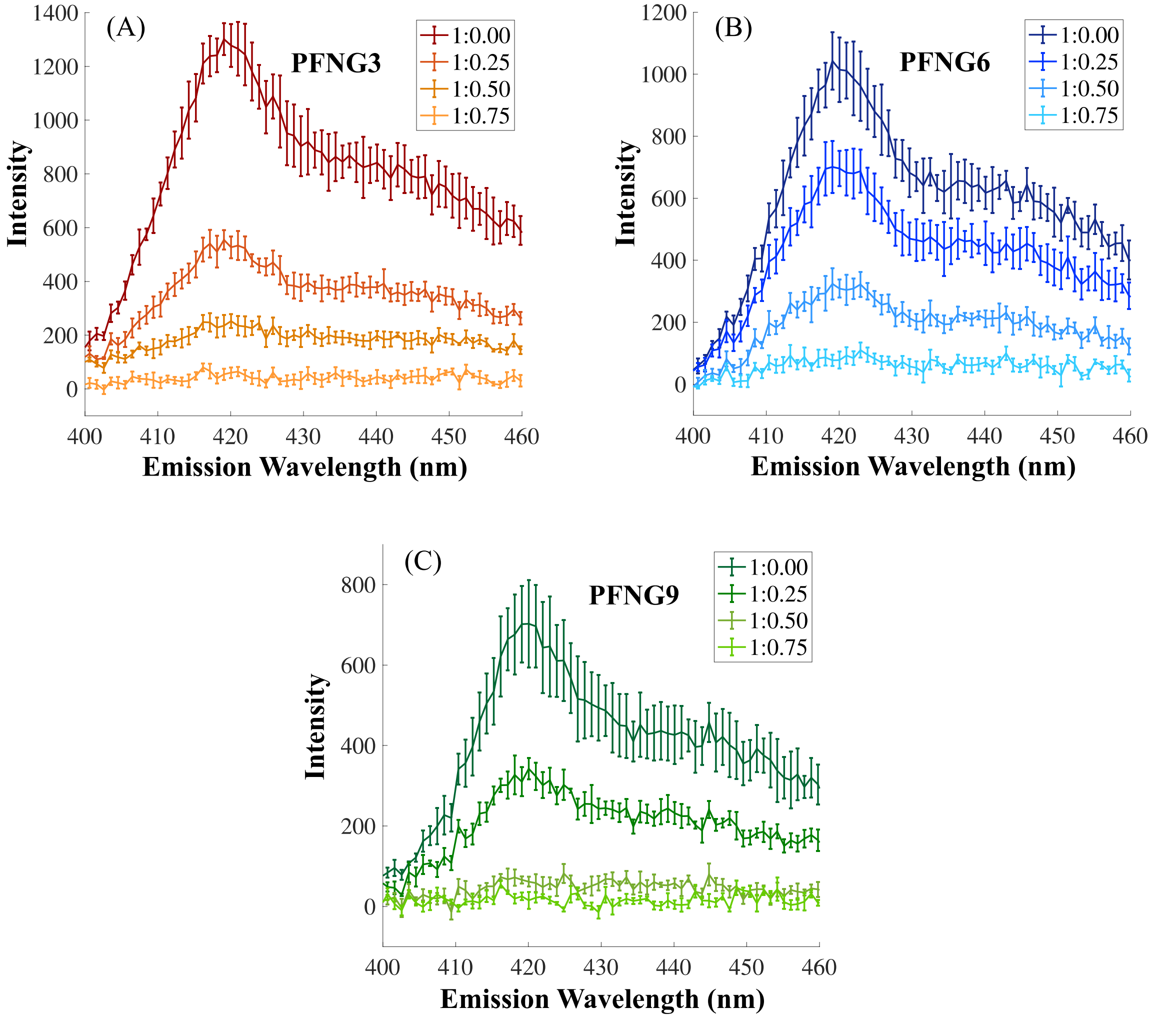

Figure S31: PL spectra of the PFNGX polymers at the same fixed molar concentration in the presence of varying amounts of PTAK: (A) PFNG3; (B) PFNG6; (C) PFNG9. For each polymer, the PL intensity is a monotonically decreasing function of [PTAK].

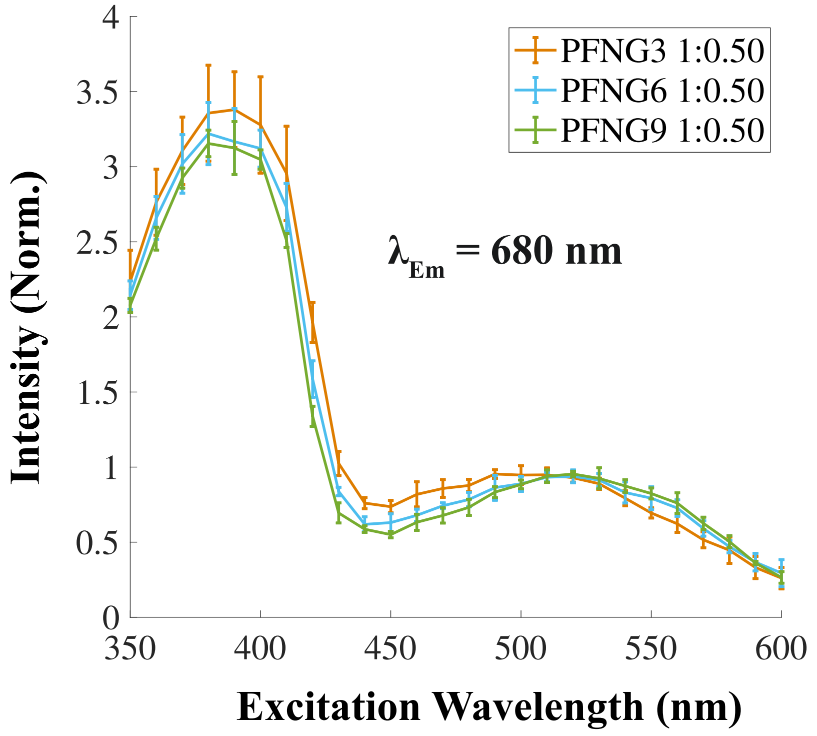

Figure S32: Photoluminescence excitation spectra of PFNGX:PTAK complexes at the 1:0.5 molar charge ratio. The emission wavelength was fixed at 680 nm.
